# A Coupled Model of Hydraulic Eco‐Physiology and Cambial Growth — Accounting for Biophysical Limitations and Phenology Improves Stem Diameter Prediction at High Temporal Resolution

**DOI:** 10.1111/pce.15239

**Published:** 2024-10-24

**Authors:** Che Liu, Mikko Peltoniemi, Pavel Alekseychik, Annikki Mäkelä, Teemu Hölttä

**Affiliations:** ^1^ Department of Forest Sciences, Faculty of Agriculture and Forestry University of Helsinki Helsinki Finland; ^2^ Institute for Atmospheric and Earth System Research (INAR) University of Helsinki Helsinki Finland; ^3^ Natural Resources Institute Finland (Luke) Helsinki Finland

**Keywords:** cambial growth, eco‐physiological modelling, photosynthesis, sink activity, soil water, stomatal behaviour, temperature, transpiration

## Abstract

Traditional photosynthesis‐driven growth models have considerable uncertainties in predicting tree growth under changing climates, partially because sink activities are directly affected by the environment but not adequately addressed in growth modelling. Therefore, we developed a semi‐mechanistic model coupling stomatal optimality, temperature control of enzymatic activities and phenology of cambial growth. Parameterized using Bayesian inference and measured data on *Picea abies* and *Pinus sylvestris* in peatland and mineral soils in Finland, the coupled model simulates transpiration and assimilation rates and stem radial dimension (SRD) simultaneously at 30 min resolution. The results suggest that both the sink and phenological formulations with environmental effects are indispensable for capturing SRD dynamics across hourly to seasonal scales. Simulated using the model, growth was more sensitive than assimilation to temperature and soil water, suggesting carbon gain is not driving growth at the current temporal scale. Also, leaf‐specific production was occasionally positively correlated with growth duration but not with growth onset timing or annual cambial area increment. Thus, as it is hardly explained by carbon gain, phenology itself should be included in sink‐driven growth models of the trees in the boreal zone and possibly other environments where sink activities and photosynthesis are both restrained by harsh conditions.

## Summary statement

A novel eco‐physiological model was constructed by incorporating formulations of growth‐related sink activities, effects of environmental annual cycle and tree hydraulics. The model emphasizes sink processes as drivers of growth as well as effects of phenological regulation in harsh environments.

## 1 Introduction

### 1.1 Sink‐Driven Tree Growth Modelling

Tree growth modelling has been developing for a long time and is valuable for ecology and economy. On the annual scale, the models commonly employ carbon analysis, which regards the growth of tree compartments as carbon fluxes and tracks carbon sequestration, allocation and turnover (McMurtrie and Wolf 1983; Mäkelä 1997; Mäkelä and Valentine 2020). The growth‐related environmental factors and processes are represented as modifiers of carbon fluxes, for example, temperature, water and irradiance (Peltoniemi et al. 2015; Tian et al. 2020), competition for light (Valentine et al. 2000; Härkönen et al. 2010) and nutrient uptake (Mäkelä et al. 2022). Such models also serve as sub‐models of larger‐scale dynamic vegetation modelling (DVM), and thus DVM is often focussed on carbon sequestration and allocation and coupled with annual gross primary production (GPP) (e.g., Bonan et al. 2011; Haverd et al. 2018; but see Leuzinger et al. 2013).

Despite the good performance of these models on historical yearly‐scale data, the focus on the ‘carbon source pathway’, that is, modelling environmental effects on cambial growth through carbon sequestration and allocation has been challenged, especially at finer temporal resolution (e.g., sub‐monthly; Körner 2015; Fatichi et al. 2019). Correspondingly, calls have been made in recent years for more explicit expression of direct environmental effects on sink activities (Fatichi et al. 2019; Friend et al. 2019) for the following reasons. First, most experimental observations at the ecosystem level suggest that cambial growth is limited by environmental constraints on sink activities rather than carbon availability (Millard, Sommerkorn, and Grelet 2007). In a global view, annual GPP has a correlation of only < 0.40 with tree ring widths between geographically and climatically similar sites (Cabon et al. 2022). Second, there is a great divergence in predicting the future vegetation under changing climates using DVM driven by carbon sources (Friedlingstein et al. 2014). This divergence can be considerably explained by the direct environmental effects on wood formation (Friend, Eckes‐Shephard, and Tupker 2022), and thus sink‐driven xylogenesis modelling has been suggested to be incorporated into growth models (Eckes‐Shephard et al. 2022).

It has long been known that temperature and turgor pressure are the key factors affecting the sink activities of cambial growth (Lockhart 1965; Körner 2015; Steppe et al. 2015; Peters et al. 2021; Potkay et al. 2022). Upon such understanding, attempts have been undertaken to link cambial growth to vasculature functioning on the whole‐tree scale (e.g., Steppe et al. 2006; De Schepper and Steppe 2010; Holtta et al. 2010; Peters et al. 2021; Potkay et al. 2022). Also, enzymatic metabolism has been formulated as a function of temperature and water potential based on biochemical analysis (Johnson, Eyring, and Williams 1942; Parent et al. 2010; Cabon et al. 2020). Nevertheless, challenges and uncertainties still exist in applying such models to biomes with low photosynthetic production and low growth rates (GR), for example, boreal forests. In boreal forests, both photosynthesis and cambial growth of trees are heavily impacted by the annual cycles of environmental factors, including temperature, water conditions and radiation (Pelkonen and Hari 1980; Jarvis and Linder 2000; Hari and Makela 2003; Mäkelä et al. 2008; Yun et al. 2018), and the cumulative effects of such annual cycles are represented by phenology (Kramer, Leinonen, and Loustau 2000; Delpierre, Vitasse, et al. 2016; Hänninen 2016). Previous field studies have hinted at connections between cambial phenology and carbon source limitation. For example, under prolonged constraints on carbon gain, the GR of boreal conifers decline as their maintenance respiration rates and carbon allocation to storage are maintained or increased (Huang et al. 2021). Thus, low photosynthetic production may result in reduced and delayed growth, which indeed occurs in boreal forests (net primary production [NPP] < 500 g C m^−2^ year^−1^; Cramer et al. 1999). Also, stored soluble sugars help boreal trees defend against frost damage in early spring and increase their resilience (Hartmann and Trumbore 2016; D'Andrea et al. 2021), which is a crucial factor of physiological recovery from wintry conditions and growth onset (Linkosalo, Hakkinen, and Hanninen 2006; Begum et al. 2013). Furthermore, modelling studies have found that nonstructural carbon (NSC) storage is closely correlated with growth duration (Schiestl‐Aalto et al. 2015; Cartenì et al. 2023). Therefore, phenology has been taken in a rich literature as a key intermediate variable in modelling boreal trees' growth, photosynthesis and their interaction (e.g., Hänninen and Hari 2002; Richardson et al. 2013; Delpierre, Vitasse, et al. 2016; Gennaretti et al. 2017; Bowling et al. 2024). Nevertheless, the incorporation of cambial phenology into the biophysical formulation of sink activities is yet to be tested, and the phenology's representativity of carbon gain in such a framework is yet to be analysed as well.

### 1.2 Modelling Against Point Dendrometer Observations and Parameterization

Compared with growth modelling on large temporal scales (typically annual), growth at high temporal resolution has been modelled much less, while sink functionality is particularly important on such finer scales (Fatichi et al. 2019). Recent studies have developed mechanistic models of cambial growth on a daily scale (e.g., Chan et al. 2016; Mencuccini et al. 2017), facilitated by observations using the point dendrometer. The point dendrometer or linear displacement transducer (e.g., Deslauriers, Rossi, and Anfodillo 2007) has become a widely used device for measuring dynamics of stem radial dimension (SRD) at high precision (on the scales of µm and minutes). However, it is not straightforward to disaggregate the recorded SRD dynamics into two components, namely hydraulic fluctuations (reversible) and growth (irreversible). Methods have been proposed to estimate either growth or hydraulic fluctuations first and take the other as the remainder. For example, Zweifel et al. (2016) have suggested that tree growth is highly suppressed during daytime as water potential is low and there is water deficit‐induced stem shrinkage. Therefore, they have defined cambial growth as the increment between each two temporal maxima of SRD. This separation method is easy and has been widely applied (e.g., Schäfer et al. 2019; Eitel et al. 2020; Güney et al. 2020), but it may result in questionable growth detection for example, during the winter (Zweifel et al. 2020). The alternative methods of processing dendrometer data simulate, first, the reversible expansion/contraction due to hydraulic dynamics (e.g., Chan et al. 2016; Mencuccini et al. 2017) and then estimate growth using the remaining dendrometer data after subtracting the hydraulic expansion/contraction of the stem. However, estimating hydraulic expansion/contraction using such models requires difficult parameterization and highly demanding measurements of inputs (e.g., accurate water potential in chrono‐sequence), and comprehensive models that simulate both hydraulic dynamics and growth (e.g., Steppe et al. 2006; Holtta et al. 2010) should be tested against dendrometer data over a longer period (see Peters et al. [2021] for an example on the annual scale).

Recently, Bayesian inference has introduced powerful techniques for parameter estimation and, thus, for lowering the demand for measured inputs. The estimation is initialized with parameters' prior ranges and distributions according to the knowledge before the current data and updates the distributions given the current data (Kruschke 2014). Practically, Bayesian hierarchical modelling is commonly employed, which comprises levels of process, data and (optional) parameter models (Dietze 2017). The process model formulates the scientific questions and calculates the errors between model outputs and respective observations (e.g., modelled SRD dynamics vs. dendrometer observations). The data model describes the error probabilities using probability density function(s) (PDF) of error values (often termed likelihood function). The parameter model describes the prior probability distributions of parameter values but may be omitted if the parameters are assumed identically and uniformly distributed. The total likelihood given the current data and parameter values is the product of the results of all the PDFs in the hierarchical model according to Bayes' theorem (Gelman et al. 2014). This likelihood is updated after every iteration that samples new parameter values from the prior distributions, and the parameter estimates that are corresponding to the maximum likelihood, that is, the maxima a posteriori (MAP) estimates, are adopted as the ‘optimal’ estimates. Bayesian techniques have been facilitated with efficient adaptive Markov chain Monte Carlo (MCMC) algorithms (e.g., Vrugt et al. 2009) and applied to a range of eco‐physiological studies (e.g., van Oijen et al. 2013; Bloom and Williams 2015; Minunno et al. 2019; Liu et al. 2020; Tian et al. 2020).

### 1.3 Objectives of the Study

Our main goal was to provide a novel tool for simulating hydraulic and sink‐driven growth dynamics at high temporal resolution and with easily measured inputs. Thus, our objectives were

1. Build a coupled model of stomatal behaviour and whole‐tree hydraulics (Liu et al. 2022) and cambial growth (inspired by Cabon et al. 2020), including tasks as follows
  a. Connect the sub‐models via temperature and water potential, and extend the cambial growth model to include a description of cambial phenology.
  b. Parameterize the coupled model by Bayesian inference and prior knowledge, and test the model against observations at 30 min resolution from boreal forest sites in Finland.
2. Using the model and its estimated parameters, compare the sensitivities of cambial growth and photosynthesis to temperature and soil water content.
3. Investigate the correlation between cambial growth phenology and photosynthesis and, thus, the regulating effects of source activities on growth‐related sink activities.

## 2 Model Description

The hydraulic part of the model is based on our previous whole‐tree optimal stomata model (OSM; Liu et al. 2022), which in turn was developed from the Cowan‐Farquhar‐type stomatal optimality model (Cowan and Farquhar 1977; Cowan 1982; Hari et al. 1986). It outputs water potential to an SRD model, as water potential is the hydraulic driver of both reversible SRD variations and irreversible cambial growth. The growth model inside the SRD model simulates the expansion rates of cambial cells using Lockhart (1965) formulation, which quantifies the effects of temperature on cell expansion based on the theory of enzyme metabolism (Johnson, Eyring, and Williams 1942; Parent et al. 2010; Cabon et al. 2020). The number of active cells at the division phase is modified with the Gompertz function (Cuny et al. 2013) to reflect cambial growth phenology.

Throughout this article, superscripts (O) and (M) are used for denoting observed and modelled variables, respectively.

### 2.1 Stomatal Conductance and Whole‐Tree Hydraulics

In the Cowan‐Farquhar‐type OSM, the optimal stomatal behaviour is defined to realize minimum difference between transpiration and assimilation, both summed over a given period (Cowan 1982). Hari et al. (1986) used the inverse definition of optimality, that is:

(1) ∫[A(t)−λE(t)]dt=maximum

where *A* and *E* are assimilation and transpiration rates, respectively. The current model aims at this optimality as well, as the Lagrangian multiplier λ≝∂A/∂E reflects marginal water use efficiency (MWUE) in eco‐physiology. The solution of the steady‐state stomatal conductance (m s^−1^) for CO_2_ is (Mäkelä 1997; Mäkelä et al. 2004):

(2) $$g_{\sigma }^{*}=\left(\sqrt{\frac{C_{a}-R{\left(\frac{\iota \gamma I}{\iota I+\gamma }\right)}^{-1}}{1.6\lambda D}}-1\right)\frac{\iota \gamma I}{\iota I+\gamma }$$

where *C*_a_ is the atmospheric CO_2_ concentration (mol m^−3^; Table 1), *D* water vapour pressure deficit (VPD, mol m^−3^), *λ* or MWUE in mol CO_2_ mol^−1^ H_2_O, *R* respiration rate (mol CO_2_ m^−2^ leaf) and *ι* (m^3^ mol^−1^) and *γ* (m s^−1^) (Table 2) are the initial slope and saturation of stomatal reaction to photosynthetic photon flux density (PPFD, *I*). In the current work, we introduced a constant minimum conductance ($g_{0}$) due to incomplete closure of stomata (Duursma et al. 2019), and thus leaf‐to‐air conductance becomes:

(3) $$g_{\sigma }\left(t\right)=\max\left\{g_{0},g_{\sigma }^{*}\left(t\right)\right\}$$

which was used in the modelled transpiration rate (mol H_2_O m^−2^ leaf).

(4) $$E^{\left(M\right)}=1.6g_{\sigma }D$$

**Table 1:** Constants, their values and units.

	Meaning	Value and unit	Source
*C*_a_	Atmospheric CO_2_ concentration	1.753 × 10^−2 ^mol m^−3^ (415 ppm)	Measured average
$g_{0}$	Minimum stomatal conductance	PS: 4.5525 × 10^−5 ^m s^−1^ MS: 7.5 × 10^−5 ^m s^−1^	Heinsoo and Koppel (1999), Hari and Mäkelä (2003)
*R*_0_	Respiration rate (*R*) at 0°C	9.1 × 10^−8 ^mol CO_2_ m^−2^ leaf s^−1^	Mäkelä et al. (2004)
*Q*_10_	Increase of *R* per 10°C relative to *R*_0_	2.3	Mäkelä et al. (2004)
*S*_0_	Threshold of photosynthetic acclimation of foliage to temperature (*S*)	−4.5°C	Mäkelä et al. (2004)
*τ*_*S*_	Time constant of *S*	12 days	Mäkelä et al. (2004)
*k*_0_	Base‐case soil‐to‐leaf conductance	2.22 × 10^−8 ^mol H_2_O m^−2^ leaf s^−1^ Pa^−1^	Nikinmaa et al. (2013), Liu et al. (2022)
*θ*_sat_	Saturated soil water potential	PS: 0.918 m^3^ m^−3^ MS: 0.57 m^3^ m^−3^	Päivänen (1973), Duursma et al. (2008)
$\psi_{e}$	Air entry water potential	−745 Pa	Duursma et al. (2008)
$\psi_{l}^{\min}$	Minimum leaf water potential	PS: −3 MPa MS: −2 MPa	Martínez‐Vilalta et al. (2009), González‐Muñoz et al. (2018)
*l*_rb_	Vertical distance between the average depth of active fine roots and breast height	1.5 m	Estimated
$\Delta \psi_{g}$	Gravitational water potential difference between roots and breast height	0.01473 MPa	Product of density of water (1000 kg m^−3^), gravitational acceleration in Finland (9.82 m s^−2^) and *l*_rb_
$J_{\text{bin}}^{\max}$	Maximum water uptake rate through the bark	2 × 10^−5 ^mm s^−1^	Gimeno et al. (2022)
*k*_bc_	Bark‐to‐cambium conductance	3.643 × 10^−5 ^mm MPa^−1^ s^−1^	Mencuccini et al. (2013)
$E_{b}$	Modulus of elasticity (MOE) of the bark	15.48 MPa	Mencuccini et al. (2013)
*d*_c_	Radial diameter of a cambial cell	PS: 0.035 mm MS: 0.0325 mm	Sarén et al. (2001), Mäkinen and Hynynen (2012)
$N_{c}^{\max}$	Maximum number of active cambial cells	8.85	Cabon et al. (2020)
*a*	Scaling coefficient in the correlation between the relative cell expansion rate and temperature	5.3544 × 10^12 ^K^−1^	Cabon et al. (2020)

*Note:* In value and unit, PS and MS are peatland (Ränskälänkorpi, Norway spruce) and mineral‐soil (Hyytiälä, Scots pine) sites, respectively, if values differ. The maximum water uptake rate through the bark presented by Gimeno et al. (2022) was *c*. 2.55 × 10^−6 ^mol s^−1^, which was converted to the current $J_{\text{bin}}^{\max}$ using $J_{\text{bin}}^{\max}=\frac{2.55\times 10^{-6}M_{w}}{\rho_{w}A_{b}}\times 1000$, where the bark area (*A*_b_, m^2^), molar mass (*M*_w_, kg mol^−1^) and density (*ρ*_w_, kg m^−3^) of water (labelled with deuterium) are available from the same source.

**Table 2:** Prior ranges of estimated parameters of the whole‐tree optimal stomata model (OSM) and the stem radial dimension (SRD) model.

	Meaning	Prior minimum, maximum	References
*ξ*_m*i*,*a*_	Multiplier (*ξ*_m_, mol H_2_O m^−2^ leaf s^−1^ Pa^−1^) and power (*ξ*_p_) of the correlation between soil‐to‐root conductance and soil water content with no waterlogging	0.02, 0.25	Duursma et al. (2008), Liu et al. (2022)
*ξ*_p_		5, 15	
*η*_m*i*_	Similar to *ξ*_m_ and *ξ*_p_ but under waterlogging	0.03, 0.5	Liu et al. (2022)
*η*_p_		1.5, 10	
σ*	Optimal water table depth (cm)	20, 35	Estimated
*z*_0*a*_	Intercept (*z*_0_) and slope (*z*_1_) of the correlation between *λ* and soil‐to‐leaf conductance	−7, −2	Liu et al. (2022)
*z*_1*a*_		−1, −0.2	
*c*_*i*,*a*_	Slope of foliage acclimation to temperature regarding photosynthesis (m^3^ (mol °C)^−1^)	PS: 0.04, 0.15 MS: 0.01, 0.10	Liu et al. (2022)
*γ*	Saturation level of PPFD reaction in stomatal conductance (mm s^−1^)	PS: 1.2, 4 MS: 1.6, 4	Mäkelä et al. (2004) Liu et al. (2022)
*β*	Slope of the correlation between the sap flow‐transpiration time lag and tree height (min m^−1^)	1, 9.5	Liu et al. (2020)
$E_{i,a}$	Modulus of elasticity of wood (MPa)	PS: 300, 1000 MS: 600, 1500	Nobel (2020)
$\psi_{0}$	Thresholds in water potential $(\psi_{0}$, MPa) and temperature (*T*_0_, K) of cambial growth	−1.1, −0.3	Cabon et al. (2020)
*T*_0_		4, 10	Rossi et al. (2007)
Δ*Η*_A_	Enthalpy of activation of enzymatic system (J mol^−1^)	−9.2 × 10^4^, −5.4 × 10^4^	Parent et al. (2010)
Δ*Η*_D_	Enthalpy (Δ*Η*_d_, J mol^−1^) and entropy (Δ*S*_d_, J mol^−1^ K^−1^) differences between enzymatic system's catalytically active and inactive states	1.80 × 10^5^ 5.25 × 10^5^	Parent et al. (2010)
Δ*S*_D_		600, 1500	
$\phi_{i,a}^{\max}$	Maximum radial extensibility of cambial cell (MPa^−1^ h^−1^)	4 × 10^−3^, 17 × 10^−3^	Cabon et al. (2020)
*b*_*i*,*a*_	Displacement (*b*) and inverse rate (*τ*_G_, h) coefficients of the differentiated Gompertz function describing the number of active cambial cells	2.5, 11	Estimated
*τ*_G*i*,*a*_		200, 600	

*Note:* Subscript *i* and *a* in a symbol denote that the parameter was evaluated specifically for each tree and for each year (when multiple years of data were available), respectively. PS and MS mean the same as in Table 1. Prior maximum of E of #7 was tuned up to 1500 MPa as its dendrometer records showed considerably lower magnitudes in day‐to‐day changes. The priors of E were higher at MS for representing the full DBH of wood that the dendrometers measured cf. RBH at PS.

The modelling of *R* and *ι* in Equation 2 remains the same as in Liu et al. (2020), which are

(5) $$R=R_{0}Q_{10}^{T_{l}/10}=R_{0}Q_{10}^{\left(T+1500I\right)/10}$$

(*T*_l_, leaf temperature, calculated using air temperature [*T*] and PPFD [Kolari et al. 2007]; *Q*_10_, relative increase of *R* per 10°C; and *R*_0_, value of *R* at 0°C; *Q*_10_ and *R*_0_ are constants),

(6) $$\iota =\max\left\{c\left(S-S_{0}\right),0\right\}$$

where *c* is an estimated slope coefficient multiplied with photosynthetic acclimation to temperature (*S*) when over a threshold (*S*_0_), and the dynamics of *S* follow:

(7) $$\dot{S}\left(t\right)=\frac{T_{l}\left(T\left(t\right),I\left(t\right)\right)-S\left(t\right)}{\tau_{S}}$$

(*τ*_*S*_, time constant of *S*; *T*_l_, leaf temperature calculated as in Equation 5; the dot notation denotes the derivative with respect to and only to time, e.g., $\dot{S}≝dS\left(t\right)/dt$, which is used throughout this article). The ‘S’‐model has been found critical to reflect the photosynthetic acclimation of boreal trees (Mäkelä et al. 2004; Mäkelä et al. 2008; Schiestl‐Aalto et al. 2015).

Although the Cowan‐Farquhar‐type OSM has been widely applied and tested (Medlyn et al. 2011), a constant *λ* (MWUE) does not hold when environmental factors change significantly (Sperry et al. 2017; Dewar et al. 2018; Potkay and Feng 2023). With accounting for the effects of vasculature transport on stomatal behaviour, *λ* has been found negatively correlated with soil‐to‐leaf conductance (*k*_sl_) under optimized stomatal conductance (Manzoni et al. 2011; Sperry et al. 2017; Dewar et al. 2018). This correlation can be approximated numerically as (Hölttä et al. 2017):

(8) $$\mathrm{lg}\lambda =z_{0}+z_{1}\mathrm{lg}\left(\frac{k_{\text{sl}}}{k_{0}}\right)$$

where *z*_0_ and *z*_1_ (both negative) are estimated coefficients, *k*_0_ the reference xylem conductance, and

(9) $$k_{\text{sl}}^{-1}=k_{\text{sr}}^{-1}+k_{\text{rl}}^{-1}$$

where the soil‐to‐root conductance (*k*_sr_) was modelled depending on site condition. In mineral soils with low to medium water contents (SWC, *θ*; measured, see Section 3.2 Data Collection), *k*_sr_ is positively correlated with *θ* (Duursma et al. 2008; Carminati and Javaux 2020), which can be expressed with combined coefficients as:

(10) $$k_{\text{sr}}^{+}=\xi_{m}{\left(\frac{\theta }{\theta_{\text{sat}}}\right)}^{\xi_{p}}$$

where *ξ*_m_ and *ξ*_p_ are tree‐ or tree‐year‐specific (if multi‐year data available) parameters related to fine root properties and soil structure, and *θ*_sat_ saturated SWC. In drained peatland where SWC is typically high with regular waterlogging, $k_{\text{sr}}\left(\theta \right)$ also includes the negative effect of waterlogging due to deficient oxygen as (Liu et al. 2022):

(11) $$k_{\text{sr}}^{-}=\eta_{m}{\left(\frac{2\theta^{*}-\theta }{\theta_{\text{sat}}}\right)}^{\eta_{p}}$$

where *η*_m_ and *η*_p_ are coefficients, and θ* optimal SWC. Combining Equations 10 and 11 with a stable *k*_sr_ in between (Liu et al. 2022),

(12) $$k_{\text{sr}}^{\text{peat}}\left(\theta \left(\sigma \right)\right)=\left\{\begin{array}{lll} k_{\text{sr}}^{+}\left(\theta \left(\sigma \right)\right) & , & \sigma <\sigma^{*}-15\\ k_{\text{sr}}^{-}\left(\theta \left(\sigma \right)\right) & , & \sigma >\sigma^{*}+15\\ \frac{1}{2}\left(\max\left\{k_{\text{sr}}^{+}\left(\theta \left(\sigma \right)\right)\right\}+\max\left\{k_{\text{sr}}^{-}\left(\theta \left(\sigma \right)\right)\right\}\right) & , & \text{otherwise} \end{array}\right.$$

where *θ*(*σ*) is SWC in the peat calculated from water table depth (WTD, *σ*, in cm) using Van Genuchten (1980) model calibrated by Leppä et al. (2020) for Finnish peatlands.

(13) $$\theta \left(\sigma \right)=\theta_{\text{res}}+\frac{\theta_{\text{sat}}-\theta_{\text{res}}}{{\left[1+{\left(0.072\;\sigma \right)}^{1.371}\right]}^{1-1.371^{-1}}}$$

where *θ*_res_ is the residual SWC. The optimal WTD in Equation 12, σ*, is an estimated parameter (Table 2).

For both peatland and mineral‐soil sites, the root‐to‐leaf conductance (*k*_rl_ in Equation 9) of each sample tree *i* was estimated as (Martínez‐Vilalta et al. 2009):

(14) $$k_{\text{rl},i}\left(t\right)=\frac{\max_{t}\left\{J_{i}^{\left(O\right)}\left(t\right)\right\}}{\rho_{i}∆\psi_{\text{sl}}^{\max}}$$

where $J_{i}^{\left(O\right)}$ is observed sap flow density of tree *i* (at breast height; mol H_2_O m^−2^ sapwood s^−1^), *ρ* all‐sided leaf‐to‐sapwood area ratio (m^2^ m^−2^; year‐specific when study period is multiple years; Table 3) and the maximum leaf water potential difference between soil and leaf ($∆\psi_{\text{sl}}^{\max}$) is species‐specific.

**Table 3:** Information on the sample trees at (a) Ränskälänkorpi (peatland site, Norway spruce) and (b) Hyytiälä (mineral‐soil site, Scots pine), Finland.

(a)						
	**Plot treatment**	**Age in 2021 (yr)**	**DBH (cm)**	**Height (m)**	**Sapwood area (10^−3^ m^2^)**	**All‐sided leaf area (m^2^)**
1	Control	78	28.0	22.2	30.16	291.53
2	Control	74	16.6	15.4	8.77	99.68
3	Control	43	13.4	14.4	6.56	60.04
4	Control	58	12.1	13.6	7.76	47.08
5	Harvest	76	20.4	19.7	18.58	153.33
6	Harvest	87	26.1	23.3	30.00	252.08
7	Harvest	51	21.3	19.2	23.36	169.32
8	Harvest	53	14.3	15.9	10.39	69.12
9	Harvest	47	9.5	10.9	3.77	26.85
10	Harvest	60	17.3	18.8	16.71	106.02
11	Harvest	49	11.1	12.2	7.13	38.81

(b)					
**Name**	**Study period**	**DBH (cm)**	**Height (m)**	**Sapwood area (10^−3^ m^2^)**	**All‐sided leaf area (m^2^)**
Pentti	2015−2019	22.3−23.8	17.9−19.2	24.10−26.12	77.54−87.54
Sylvi	2015, 2017−2019	21.8−22.9	18.4−19.7	23.43−24.92	73.87−81.12

*Note:* The properties of trees are as of late 2020 (except tree age) in (a) and displayed with the values at the beginning and the end of the study period in (b). The exact age of the trees at Hyytiälä was unknown, whereas the stand emerged in 1960s.

Abbreviation: DBH, diameter at breast height.

### 2.2 Wood Expansion and Formation

The dynamics of SRD (*d*) include elastic changes (reversible) and growth (irreversible) (e.g., Chan et al. 2016), that is:

(15) $$\dot{d}={\dot{d}}_{\text{ela}}+{\dot{d}}_{\text{gro}}$$

**Figure 1:**
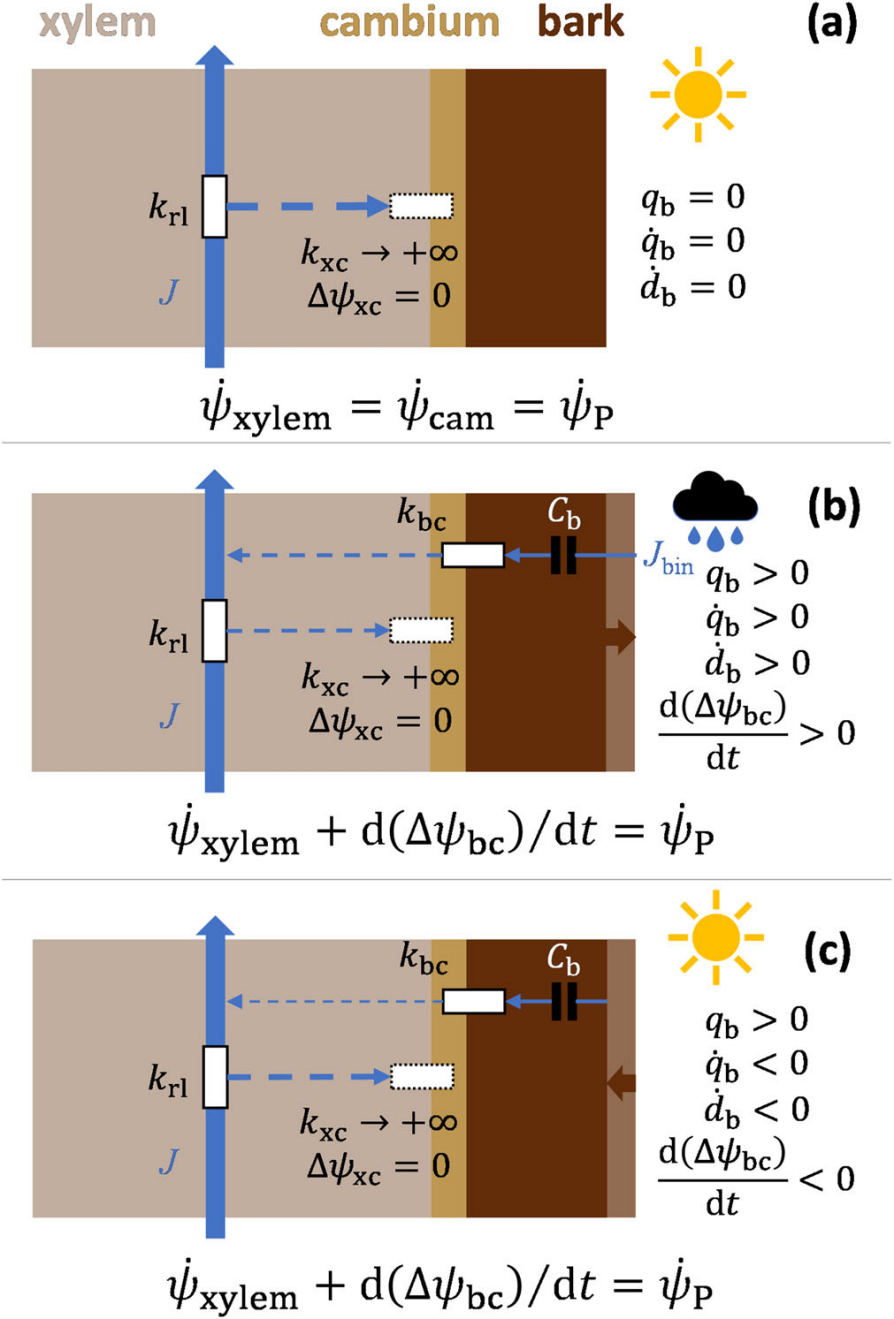
Schematic diagram of modelling the hydraulic properties of the tree trunk at breast height when the bark is fully and constantly dry (a), absorbing rainwater (b) and drying out after a rain event (c). The conductance between xylem and cambium was always (a−c) assumed to be infinitely large (k_xc_ → +∞), and thus the potentials of xylem and cambium are instantly equilibrated ($∆\psi_{\mathrm{xc}}=0)$. Hence, water flows between xylem and cambium (dashed blue arrows) not explicitly expressed in the model. Cambial osmotic potential was assumed constant, and thus, the dynamics of xylem water potential and cambial turgor pressure (${\dot{\psi }}_{P}$) equal without rain effects (a). During (b) and after (c) rain, the bark thickness (*d*_b_) increases and decreases, respectively. During these processes, the rainwater in the bark affects ${\dot{\psi }}_{P}$ via the potential difference between bark and cambium ($∆\psi_{\mathrm{bc}}$), depending on rainwater uptake rate (*J*_bin_), the capacitance of the bark (*C*_b_) and the conductance between bark and cambium (*k*_bc_), until the bark returns to the state in (a). See the main text for the full definitions of the symbols and Equations (14), (15), (16), (17), (18), (19), (20), (21), (22), (23) for the full formulation of wood expansion and formation.

The driver of ${\dot{d}}_{\text{ela}}$ is the dynamics of turgor pressure (at breast height in this study; ${\dot{\psi }}_{P}$), following:

(16) $${\dot{d}}_{\text{ela}}=\frac{{\dot{\psi }}_{P}}{E}d_{0}$$

where *d*_0_ is the value of *d* at a reference hydration status (here defined as *d* at the beginning of stem cambial growth), and E is the modulus of elasticity (MOE) or Young's modulus of the bulk stem (including wood and the inner bark). As we focussed on the radial growth in the current study, we employed the linear MOE (i.e., one‐dimensional in geometry in MPa m m^−1^, cf. volumetric in MPa m^3^ m^−3^; Nobel 2020). The linear MOE was a tree‐ and year‐specific (if multiple years of data were available) parameter (Table 2) and constant for one tree‐year (Holtta et al. 2010; Chan et al. 2016; Mencuccini et al. 2017; see Supporting Information S1: Note S1 for further discussion on assuming MOE independent of turgor). The year‐specificity was in consideration that the dendrometers were changed in location on the sample trees at the beginning of every year. When modelling ${\dot{\psi }}_{P}$, we assumed that the water potentials of xylem ($\psi_{\text{xylem}}$) and cambium ($\psi_{\text{cam}}$) were instantly equilibrated (Daudet et al. 2002; Thompson and Holbrook 2003). As $\psi_{\text{cam}}=\psi_{P}+\psi_{\Pi }+\psi_{g}$ (where $\psi_{\Pi }$ and $\psi_{g}$ are osmotic and gravitational potentials, respectively), ${\dot{\psi }}_{\text{xylem}}={\dot{\psi }}_{\text{cam}}={\dot{\psi }}_{P}$ upon constant cambial osmolality and study height and that xylem is the sole water source of cambium that is, there is no rainwater influx through the bark (Figure 1a). Assuming cambial $\psi_{\Pi }$ constant at a certain height is a common practice for simplicity in similar modelling works (e.g., Cabon et al. 2020), considering it is turgor (along with temperature) that co‐drives cell expansion (Lockhart 1965). Also, measured osmolality at the tree base has been found indeed fairly constant at our mineral‐soil site (Hyytiälä; Paljakka et al. 2017). Under these assumptions and conditions, ${\dot{\psi }}_{\text{xylem}}={\dot{\psi }}_{\text{cam}}={\dot{\psi }}_{P}$ can be calculated using soil water potential ($\psi_{s}$) and potential loss between soil and breast height due to viscous flow (i.e., sap flow divided by conductance, analogous to Ohm's law in electricity), that is:

(17) $${\dot{\psi }}_{\text{cam}}\left(t\right)={\dot{\psi }}_{P}\left(t\right)={\dot{\psi }}_{s}\left(t\right)-\frac{d}{\rho dt}\left(\frac{J^{\left(O\right)}\left(t\right)}{k_{\text{sb}}\left(t\right)}\right)$$

where *k*_sb_ is soil‐to‐tree trunk (breast height) conductance (estimated using soil‐to‐root [*k*_sr_] and root‐to‐breast height [*k*_rb_] conductances; Supporting Information S1: Methods S1), and *ρ* is to unify the units of observed sap flow density (*J* ^(O)^) and *k*_sb_ as per leaf area. When rain events occurred, however, the dendrometer readings showed considerable expansion during and contraction after the events, whereas similar dynamics were not seen in SWC or WTD. We ascribed this phenomenon to rainwater uptake through the lenticels, stored temporarily in the bark, and thus its impact on $\psi_{P}$. To address these rainwater effects, we added a new term $d\left(∆\psi_{\text{bc}}\right)/dt$ to Equation 17 to denote the dynamics of potential difference between cambium and bark (Equation 18).

The bark was regarded as a hydraulic capacitor charged with rainwater and discharged by the cambium (Figure 1b,c). We evaluated the charging influx (*J*_bin_) as a function of rain intensity (*w*_rain_), and *J*_bin_ was set not to exceed an experimentally measured value ($J_{\text{bin}}^{\max}$; Gimeno et al. 2022). Thus, see Supporting Information S1: Methods S2 for detail.

(18) $$\frac{d\left(∆\psi_{\text{bc}}\right)}{dt}={\dot{q}}_{b}\left(t;w_{\text{rain}},J_{\text{bin}}^{\max}\right)\frac{E_{b}}{d_{b0}}$$

where *q*_b_ (mm), $E_{b}$ (MPa) and *d*_b0_ (mm) are the water volume per bark area, linear MOE and initial thickness of the bark, respectively. Add Equation 18 to Equation 17,

(19) $${\dot{\psi }}_{\text{cam}}\left(t\right)={\dot{\psi }}_{P}\left(t\right)={\dot{\psi }}_{s}\left(t\right)-\frac{d}{\rho dt}\left(\frac{J^{\left(O\right)}\left(t\right)}{k_{\text{sb}}\left(t\right)}\right)+{\dot{q}}_{b}\left(t\right)\frac{E_{b}}{d_{b0}}$$

Note that $q_{b}=0$ and ${\dot{q}}_{b}=0$ when the bark is fully and constantly dry (Figure 1a). ${\dot{\psi }}_{P}$ calculated using Equation 19 was used in Equation 16 for ${\dot{d}}_{\mathrm{ela}}$.

The soil water potential was estimated for peat ($\psi_{s}^{\text{peat}}$) using Van Genuchten (1980); Hallema et al. (2015):

(20) $$\psi_{s}^{\text{peat}}\left(\theta \right)=-{\left[\frac{{\left(\frac{\theta -\theta_{\text{res}}}{\theta_{\text{sat}}-\theta_{\text{res}}}\right)}^{-4.4248}-1}{0.0231}\right]}^{0.774}\left(\text{cm}\right)\times 98.2\;(\text{Pa}\;{\text{cm}}^{-1})$$

where the water content *θ* was estimated using Equation 13, and its saturation (*θ*_sat_) and residual (*θ*_res_) points had the same values as in Equation 13. For mineral soils, the water potential is:

(21) $$\psi_{s}^{\text{mineral}}=\psi_{e}{\left(\frac{\theta }{\theta_{\text{sat}}}\right)}^{\frac{3-\xi_{p}}{2}}$$

where $\psi_{e}$ is the air entry water potential, and the other symbols are the same as in Equation 10 (Duursma et al. 2008).

Growth dynamics of one radial file (${\dot{d}}_{\text{gro}}$, mm) were modelled based on quantifying the expansion‐driven division of cambial cells that produces new wood cells along that direction. The cambial cells were assumed to double their volume before division (Cabon et al. 2020) and to have an equal expansion rate on all three dimensions, and thus ${\dot{d}}_{\text{gro}}$ was modelled as:

(22) $${\dot{d}}_{\text{gro}}=N_{c}\frac{p}{\ln2}{\overset{®}{d}}_{c}$$

where *N*_c_ is the number of active cambial cells in one radial file, *p* the relative linear expansion rate of the cells ($p≝{\dot{d}}_{c}/d_{c}$; h^−1^; Supporting Information S1: Methods S3), and, for simplicity, the average radial diameter of a mature tracheid (${\overset{®}{d}}_{c}$) is used for converting new cell number to new wood thickness. *p* is determined by turgor ($\psi_{P}$) and temperature (*T*, K) (Lockhart 1965). However, to avoid introducing uncertainty by estimating osmotic potential and to utilize the prior knowledge of the water potential threshold for cambial growth (Cabon et al. 2020), we used total water potential of cambium at breast height ($\psi_{\text{cam}}$) for estimating *p*. $\psi_{\text{cam}}$ was estimated as:

(23) $$\psi_{\text{cam}}\left(t\right)=\left[\psi_{s}\left(t\right)-\frac{J^{\left(O\right)}\left(t\right)}{k_{\text{sb}}\left(t\right)}+\frac{E_{b}}{d_{b0}}\int {\dot{q}}_{b}\left(t\right)dt\right]-∆\psi_{g}$$

That is, the antiderivative of Equation 19 with the gravitational potential difference between roots and breast height ($∆\psi_{g}$) subtracted, and *p* is

(24) $$p=\left\{\begin{array}{ll} \phi \left(T\right)∙\left(\psi_{\text{cam}}-\psi_{0}\right), & \psi_{\text{cam}}\geq \psi_{0}\\ 0, & \psi_{\text{cam}}<\psi_{0} \end{array}\right.$$

where $\psi_{0}$ (an estimated parameter) is the threshold water potential for growth. Note that $\left(\psi_{\text{cam}}-\psi_{0}\right)$ is still the difference in turgor as the osmolality at breast height was assumed constant. The cell linear extensibility (on the radial dimension of the trunk) *ϕ*(*T*) (MPa^−1^ h^−1^) in Equation 24 is (Johnson, Eyring, and Williams 1942; Cabon et al. 2020):

(25) $$\phi \left(T\right)=\left\{\begin{array}{ll} \phi^{\max}\frac{\mathrm{aT}\text{exp}\left(\frac{∆H_{A}}{8.3145\;T}\right)}{1+\text{exp}\left[\frac{∆S_{D}}{8.3145}\left(1-\frac{∆H_{D}}{T∆S_{D}}\right)\right]}, & T\geq T_{0}\\ 0, & T<T_{0} \end{array}\right.$$

where *a* is a scaling constant, $\psi_{0}$ and *T*_0_ are respective thresholds in water potential and temperature and Δ*Η*_A_, Δ*Η*_D_ and Δ*S*_D_ characteristics of the enzymatic system, namely, the enthalpy of activation, and the enthalpy and entropy differences between the active and inactive states, respectively. Δ*Η*_A_, Δ*Η*_D_ and Δ*S*_D_ were estimated parameters, site/species‐specific and independent of temperature (see Supporting Information S1: Note S2 for further discussion on the modelled temperature effects in Equations 24 and 25). Based on theoretical analysis of enzymatic systems (Johnson, Eyring, and Williams 1942), this carbon sink‐limited growth model has earned applicability to a wide range of plants in the temperate zone (Parent and Tardieu 2012). Nevertheless, the strong phenology in the boreal zone associated with low NPP may downregulate the growth dynamics, and thus we adopted the derivative of the Gompertz function (Cuny et al. 2013) to explicitly describe the phenology and used it for modifying *N*_c_ in Equation 22 as:

(26) $$N_{c}\left(t\right)=N_{c}^{\max}\frac{bT}{\tau_{G}}\text{exp}\left[-b\text{exp}\left(-\frac{t}{\tau_{G}}\right)-\frac{t}{\tau_{G}}\right]$$

where *b* (unitless) and *τ*_G_ (h) are displacement (delay of growth onset) and inverse rate coefficients (Table 2), respectively, and the time unity T=1h is for unifying the unit of the equation. Algebraically similar adjustment to the model of enzyme metabolism (Equation 25) can be also seen in Cabon et al. (2024).

## 3 Methods and Materials

### 3.1 Study Sites and Sample Trees

The study sites were at Ränskälänkorpi (61.2° N, 25.3° E) and Hyytiälä (61.8° N, 24.3° E) in southern Finland. Ränskälänkorpi is a nutrient‐rich and mesic peatland drained in the 1960s. The mean summer (July 1981 to 2010) temperature at the site was 16.6°C and the mean annual precipitation (1981−2010) *c*. 600 mm year^−1^. The site was dominated by Norway spruce (*Picea babies* [L.] H. Karst.) with occasional Scots pine (*Pinus sylvestris* L.) and downy birch (*Betula pubescens* Ehrh.) and sparse understorey vegetation of mosses and dwarf shrubs (Laurila et al. 2021). The peat layer thickness was *c*. 70−120 cm. The site was separated into control and selection harvest blocks of approximately the same area. The harvest was conducted in March 2021 before the growing season, rendering the basal area density of Norway spruce to be 12 versus 35.8 m^2^ ha^−1^ in the control. The site at Hyytiälä was sub‐xeric and dominated by approximately even‐age Scots pine sprouted in 1960s, where the mean summer (June and July 2009−2018) temperature at Hyytiälä was *c*. 15°C and the mean precipitation in the summer is 190.9 mm year^−1^ (Liu et al. 2020). Ränskälänkorpi and Hyytiälä are referred to as PS (peatland site) and MS (mineral‐soil site) hereafter.

Eleven Norway spruce trees and two Scots pine trees (named ‘Pentti’ and ‘Sylvi’) were selected as sample trees at PS and MS, respectively (Table 3). Having been monitored since 2015, Pentti and Sylvi were close (distance ≈ 65 m) to Station II for Measuring Forest Ecosystem‐Atmosphere Relations (SMEAR II; Hari et al. 2013). The foliage biomass of all the sample trees at both sites was estimated using measured tree height and DBH (Repola 2009). The specific leaf area used for converting biomass to an all‐sided leaf area was 9.9 m^2^ kg^−1^ dry mass for Norway spruce (Stenberg et al. 1999) and 10.64 m^2^ kg^−1^ for Scots pine (measured). The total bark (outer bark plus phloem) thickness at PS was measured with a scalpel and at MS estimated following Wilms et al. (2021).

### 3.2 Data Collection

A meteorological station has been operating at PS since 2019 in each of the control and harvest blocks, which collected data on air temperature and relative humidity. PPFD was measured from a central mast higher than the canopy. WTD was automatically recorded (Odyssey Capacitance Water Level Logger, Dataflow Systems Limited, New Zealand) within PVC tubes inserted into the ground equidistantly (*c*. 80 m) at nine locations along each of four transects co‐centred at the central mast. The SWC data used in modelling was converted from measured WTD (Leppä et al. 2020; Liu et al. 2022). During the study period, WTD ranged 4−75 cm (average 37.5 cm) and 19−53 cm (average 41.5 cm) in the control and harvest blocks, respectively.

Sap flow density was measured with heat pulse velocity sap flow and water content sensors (HPV‐06, Implexx Sense, Australia) at PS. Mounted on the trees' south side at breast height, each sensor comprises three 30‐mm‐long probes, two heated with one reference in the middle, with a 6 mm distance between each two. The sensors were covered by well‐aerated aluminium radiation shields. Temperature differences at 10 mm (inner in the xylem) and 20 mm (outer) from the probes' tips following heat pulse were recorded every 15 min and converted to sap flow rate (L h^−1^) using the built‐in method species‐specific calibration of the device. The average of the inner and outer readings was used for calculating *J* ^(O)^ per sapwood area, which was measured at breast height.

At MS, the data of the aforementioned environmental variables as well as sap flow were collected from the SMEAR system (https://smear.avaa.csc.fi/). Sap flow was measured using the thermal dissipation method (‘Granier‐type probes’; Granier, 1987), and SWC was directly measured (TDR100 Reflectometer, Campbell Scientific, Utah, USA; ML3 ThetaProbe Soil Moisture Sensor, Delta‐T Devices, UK) at the depth between 14 and 25 cm (B1 horizon). The moving average of SWC over 24 h was used to avoid outlying values. During the drought in 2018, SWC at the B1 horizon ranged only 0.10−0.35 (average 0.20) and maximum VPD *c*. 3.3 kPa (average 0.72 kPa) during the growing season. Drought also likely affected tree growth in 2019, which was a moderately dry year as well (SWC 0.13−0.45, average 0.25; VPD maximum 2.7 kPa, average 0.56 kPa).

At both sites, sample tree radial dimensions at breast height were measured by point dendrometers with pneumatic push and an accuracy of 1 µm (AX‐5, Solartron Metrology, UK). The outer bark at the locations of transducers was removed before installation, and the transducers were installed against the inner bark (phloem). At PS, the dendrometer was installed through a plate on each tree, and the plate was attached to the trees by metal rods penetrating into the heartwood. Both the plates and the rods were made of Invar (FeNi36 alloy) known for near‐zero thermal expansion, and thus the system should have recorded SRD dynamics without significant interference of ambient temperature variations. At MS, the dendrometers were fixed on the trees by rectangular steel frames (Chan et al. 2016), whose thermal expansion was taken into account at analysing the raw records, following Sevanto et al. (2005). The location of each dendrometer was changed before the beginning of each growing season. Data were rejected due to technical failure in both trees in 2018 before June and in Sylvi throughout 2016. All the data of each site were synchronized to 30 min resolution accordingly.

The beginning of the data used for OSM was the growing season onset defined as the first day of the year (DOY) with S≥0°C (Equation 6), and for SRD was DOY 132 (Jyske et al. 2014).

### 3.3 Model Parameterization

The meanings and values of the constants are displayed in Table 1. The parameters (Table 2) were estimated using a data model that describes the probability density distributions of the error matrices of OSM and SRD model (Supporting Information S1: Methods S4). The estimation was conducted by the adaptive MCMC algorithm DREAM_(ZS)_ (Vrugt et al. 2009) in R 4.2.1 (R Development Core Tea 2022) with package ‘BayesianTools’ (Hartig, Minunno, and Paul 2019). The models' 99% credible intervals were obtained also using the data model by sampling 9000 parameter vectors randomly from the posterior distribution of the calibration.

To examine the necessity of expressing cambial phenology, alternative models (AM) (1) excluding the phenology of cambial cell number (Equation 26) and (2) using only Equation 26 for simulating ${\dot{d}}_{\text{gro}}$were tested against the same data. In AM1, $N_{c}\equiv N_{c}^{\max}$ (constant at 8.85; Table 1) in Equation 22. In AM2, the mechanistic expressions of enzyme metabolism and cell expansion (Equations 24 and 25) are unused, $\left(\psi_{P}-\psi_{0}\right)$ is set constant at 1 MPa, and thus *p* (Equation 22) is replaced with $p^{\left(\text{AM}2\right)}\equiv \phi^{\max}\;\times \;(1\;\text{MPa})$. Therefore, the GR of AM2 is modelled as:

(27) $${\dot{d}}_{\text{gro}}^{\left(\text{AM}2\right)}=\frac{p^{\left(\text{AM}2\right)}}{\ln2}{\overset{®}{d}}_{c}N_{c}^{\max}\frac{bT}{\tau_{G}}\text{exp}\left[-b\text{exp}\left(-\frac{t}{\tau_{G}}\right)-\frac{t}{\tau_{G}}\right]$$

where $\phi^{\max}$ is still an estimated parameter. The rest of the model structure and parameterization method remained the same as the full model (FM).

### 3.4 Post‐Parameterization Analyses

The performance of FM was assessed using linear regression and the root‐mean‐square error (RMSE). Linear regression was performed on and *J* ^(O)^/*ρ* versus *E* ^(M)^ and *d* ^(O)^ versus *d* ^(M)^.

The relative sensitivity coefficient (RSC, dimensionless; Kacser et al. 1995) was employed to evaluate the model outputs' sensitivities to environmental factors (Objective 2). The RSC of dependent *y* to an input or parameter *x* is defined as:

(28) $${\text{RSC}}_{x}^{y}=\frac{\delta y\left(x\right)}{y\left(x\right)}/\frac{\delta x}{x}$$

where *y* is modelled GR (i.e., ${\dot{d}}_{\text{gro}}$, Equation 22; mm h^−1^) or *A*, and *x* is SWC or *T*. SWC and *T* have been found crucial for radial growth of conifers (Tolwinski‐Ward et al. 2011; Eckes‐Shephard et al. 2021; Camarero et al. 2022). The environmental variables in δy(x)/δx other than SWC and *T* were constant at the measured means. The observations of SWC and *T* presented weak correlations with each other, Pearson's correlation coefficient = −0.185 and −0.204, by linear regression *R* ^2^ = 0.034 and 0.042 at the peatland and mineral‐soil sites, respectively.

To investigate the correlation between cambial growth phenology and photosynthetic production (Objective 3), the net assimilation rate (*A*, mol C m^−2^ leaf s^−1^) of the trees was estimated as (Mäkelä 1997; Hari and Mäkelä 2003)

(29) $$A=\frac{g_{\sigma }C_{a}\frac{\iota \gamma I}{\iota I+\gamma }}{g_{\sigma }+\frac{\iota \gamma I}{\iota I+\gamma }}$$

and converted to leaf‐specific production (*P* in mol C m^−2^ leaf) by summing over certain periods for correlation analyses using Pearson's coefficient. The coefficient was calculated for (1) the onset (1%) timing of cambial growth (*t*_onset_) versus *P* accumulated by *t*_onset_ (*P*_onset_), and (2) growth duration versus *P* of the whole growing season (*P*_gs_). The correlation coefficient was also calculated for modelled annual cambial area growth versus *P*_gs_ for examining the role of carbon gain in determining growth. Considering the interannual change of carbon storage is relatively small compared with the carbon cost of current‐year growth in boreal conifers (Schiestl‐Aalto et al. 2015), we focussed on correlating current‐year assimilation and cambial growth in the analyses. Cook's distance was employed in the correlation analyses to detect outliers with the threshold set at 1 (Cook and Weisberg 1982).

All the post‐parameterization analyses were conducted in R 4.2.1.

## 4 Results

### 4.1 Model Performance

**Figure 2:**
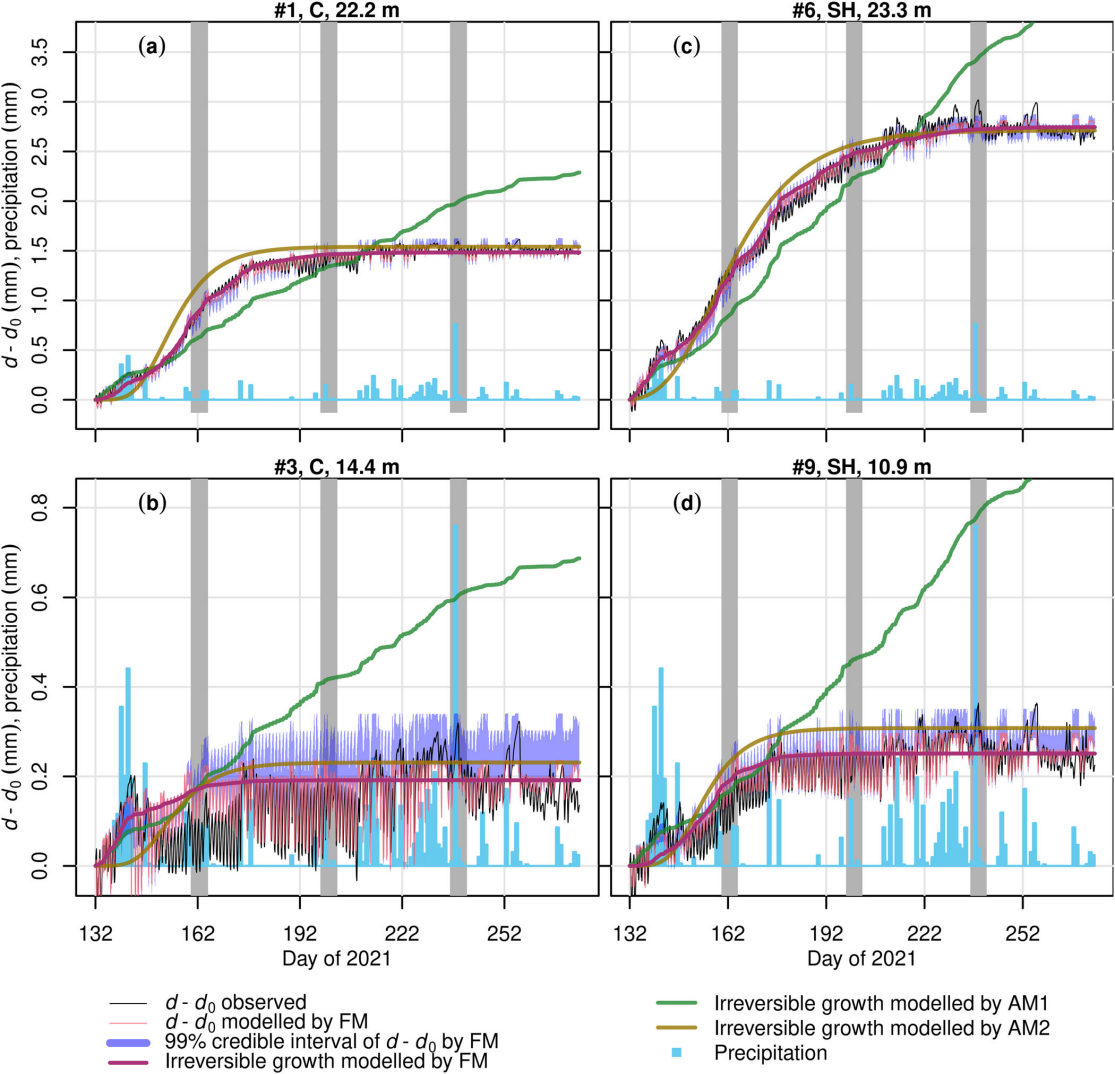
Modelled and observed SRD change in the growing season (*d*–*d*_0_) of four Norway spruce trees at the peatland site (PS). Each panel title displays the tree number, treatment of the block (C, control; SH, select harvest) and tree height. FM, AM1 and AM2 are full model, alternative models without phenology (Gompertz function) and using only the Gompertz function for modelling growth, respectively. The grey stripes, each ranging five days, correspond to the period in a zoom‐in view displayed in Figure 3. SRD dynamics due to hydraulics modelled by AMs are not shown for clarity. See Supporting Information Materials for the transpiration simulation of these trees (Supporting Information S1: Figure S1) and the results of the other Norway spruce trees (Supporting Information S1: Figure S3) at PS.

**Figure 3:**
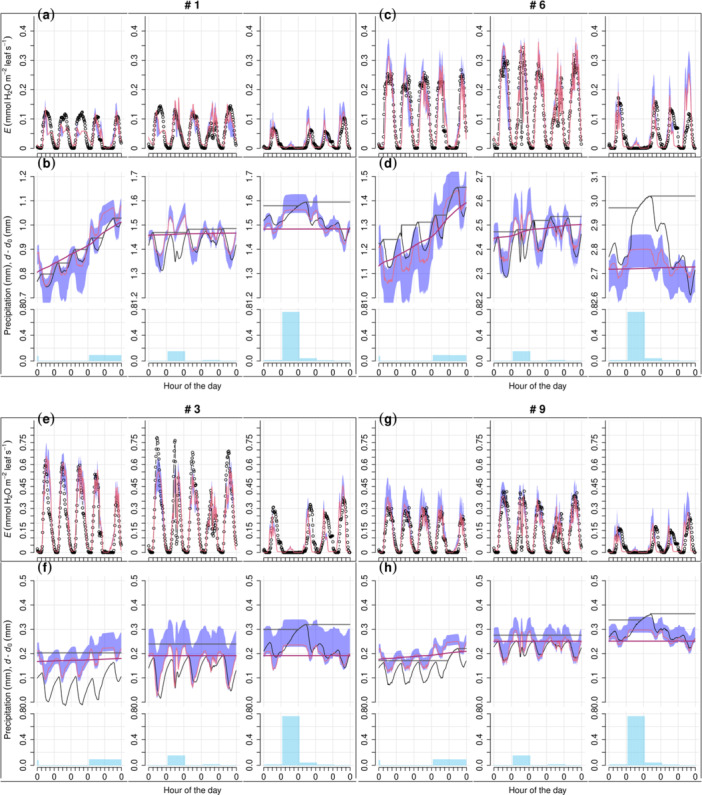
Zoom‐in view of the full model's results over selected periods at the peatland site. The displayed trees are the same as in Figure 2, and the presented 5‐day periods, corresponding to the grey stripes in Figure 2, are DOY (day of the year) 160−164, 198−202 and 236−240, respectively. The arrangement of rows and the legend are the same as in Figure 2.

**Figure 4:**
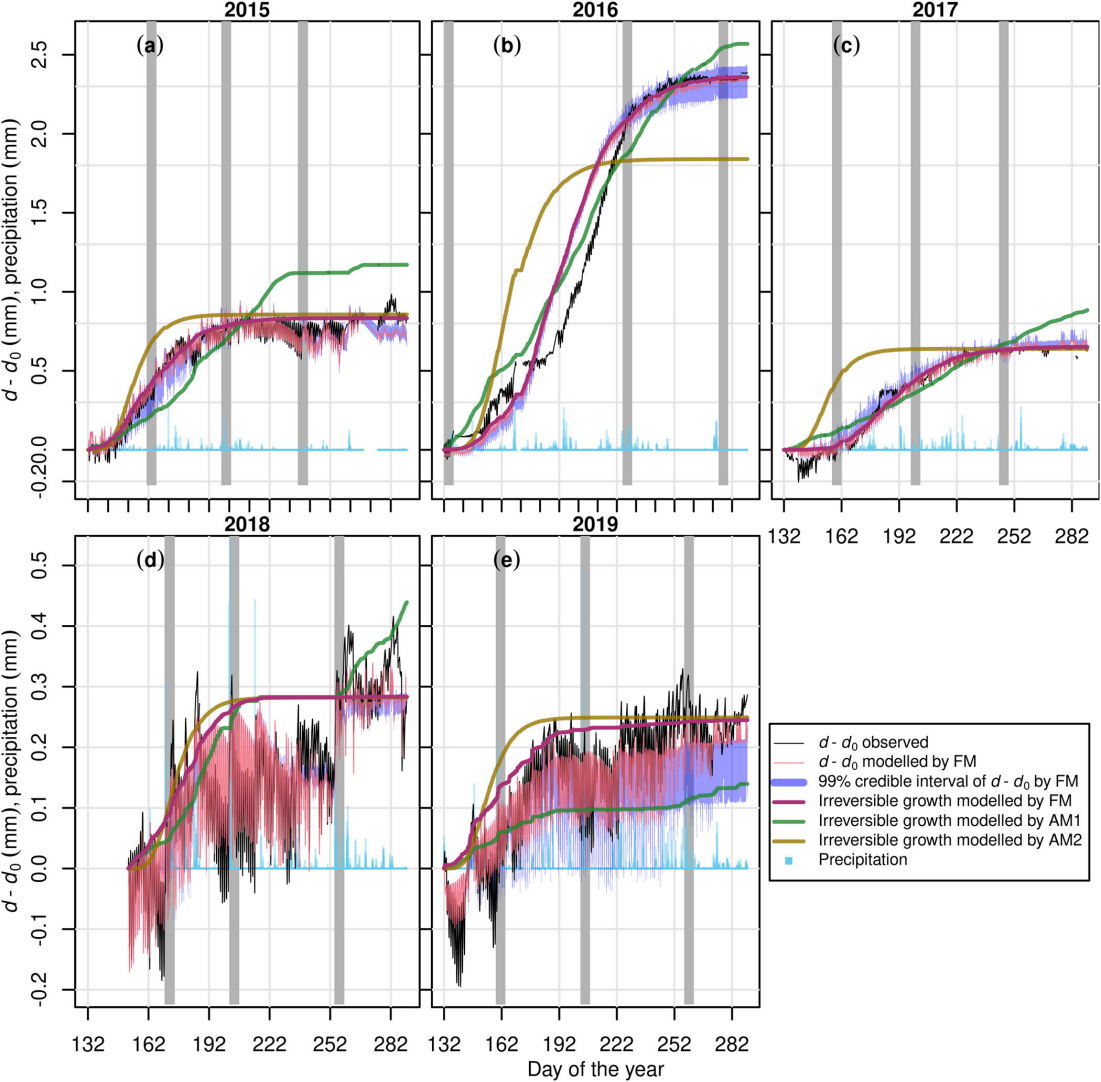
Modelled and observed SRD change in the growing season (*d*–*d*_0_) of Scots pine tree ‘Pentti’ at the mineral‐soil site (MS) over 2015−2019. The meanings of FM, AM1, AM2 and the arrangement of panels are the same as in Figure 1, except results are separated by the year (cf. tree). SRD dynamics due to hydraulics modelled by AMs are not shown for clarity. See Supporting Information Materials for the transpiration simulation of ‘Pentti’ (Supporting Information S1: Figure S2) and the results on the other Scots pine tree ‘Sylvi’ (Supporting Information S1: Figure S4) at MS.

**Figure 5:**
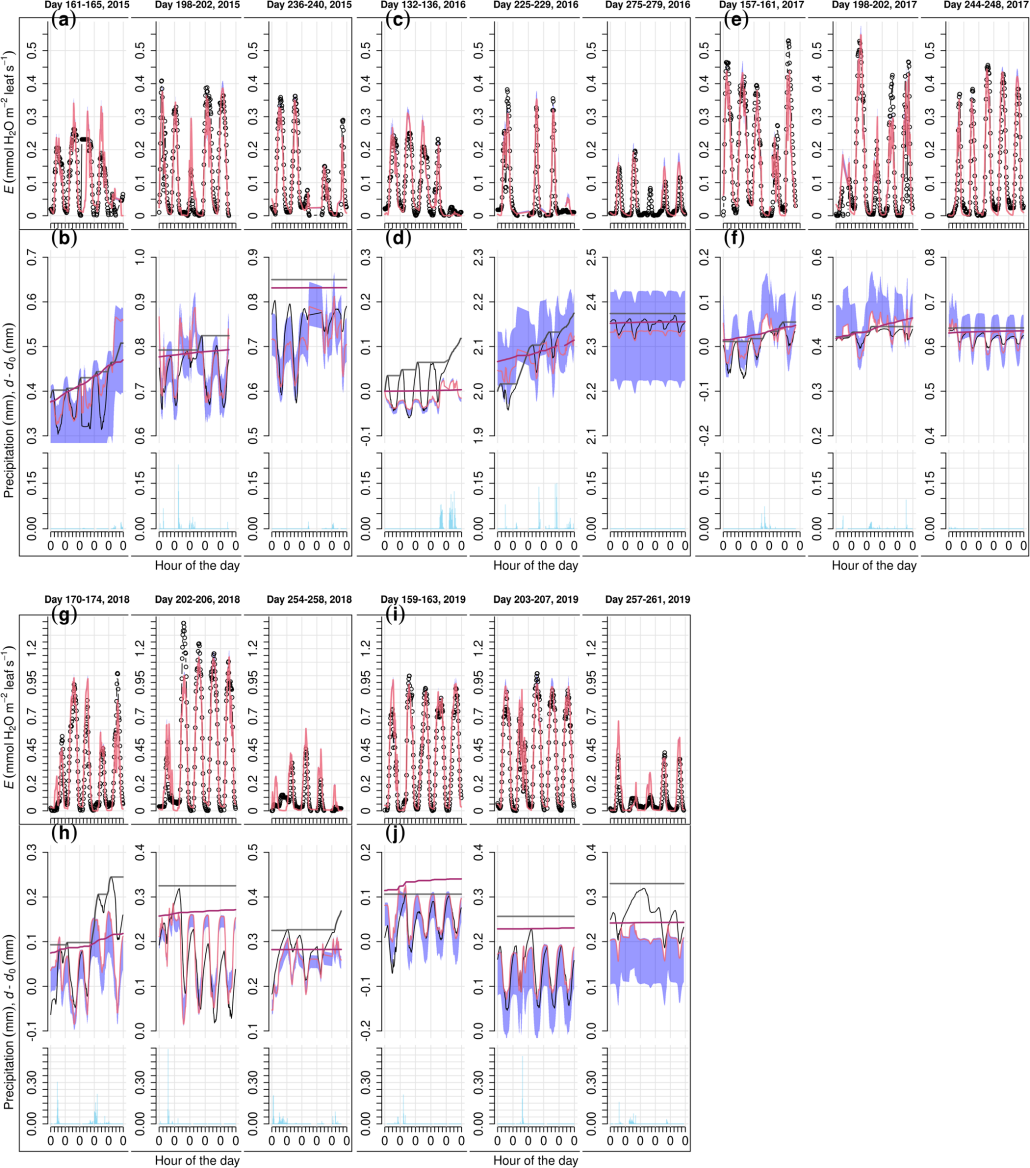
Zoom‐in view of the full model's results over selected periods on Scots pine tree ‘Pentti’ at the mineral‐soil site. Corresponding to the grey stripes in Figure 4, the presented 5‐day periods are displayed above the respective columns. The arrangement of rows and the legend are the same as in Figure 4.

The FM performed well (Figure 2, 3, 4, 5), presenting fitted slopes (FS) close to 1, minimal intercepts, *R* ^2^ > 0.97 for SRD (*d*) and *R* ^2^ > 0.76 for transpiration rate (*E*) (Table 4). On individual trees (Supporting Information S1: Table S1), the performance on *E* was of similar FS (0.83−1.05) and *R* ^2^ (0.67−0.89) for most trees/tree‐years of PS and MS, whereas the performance on *d* was worse for MS trees during the years with drought (2018 and 2019, FS > 1.2 and/or *R* ^2^ < 0.85 vs. mostly otherwise FS close to 1 and *R* ^2^ > 0.86).

**Table 4:** Site‐level summary of model performance on transpiration rate (*E*) and stem radial dimension (*d*).

	*E*		*d*	
	PS	MS	PS	MS
FS	0.916	0.939	1.00	0.973
Intercept	7.68	10.5	−5.18	4.94
*R* ^2^	0.763	0.860	0.996	0.980
RMSE	60.1	79.4	59.6	84.6

*Note:* PS and MS mean the same as in Table 2. Intercepts are in the same respective units as RMSE values. All *p* < 0.0001.

Abbreviations: FS, fitted slope of observed to modelled values; RMSE, root‐mean‐square error, in µmol m^−2^ leaf s^−1^ for *E* and µm for *d*.

For both sites, model performance at mid‐summer (DOY ≈ 200) was better, whereas occasional high errors of SRD occurred during early or late growing season (e.g., Figures 3d,f and 5b,j). The bark module (Equation 17, Supporting Information S1: Methods S1) simulated well the SRD dynamics associated with rain events, although it underestimated the expansion in early autumn (Figure 3d,f,h) and contraction in drought years (Figure 5h,j). At both sites, the modelled cumulative growth was noticeably different from that defined using Zweifel et al. (2016) method (e.g., Figures 3d and 5d,h), as the latter seems to have been affected by the SRD expansion due to rain.

In contrast with FM, both AMs failed to capture the seasonal pattern of SRD. AM1 (without the Gompertz function, i.e., without cambial growth phenology) yielded a cumulative SRD more linear than the observations and commonly underestimated SRD in early growing season while overestimated at later stage (e.g., Figures 2a,c and 4a,c). However, in years with drought (2018 and 2019) at MS (Figure 4d,e), AM1 simulation was closer to FM's than in the other years (Figure 4a−c). For most trees at PS, AM2 (using only the Gompertz function to simulate growth) generated too sigmoid a temporal pattern of growth before the saturation level, which was commonly higher than the maximum cumulative growth estimated by FM (e.g., Figure 2b,d and Supporting Information S1: Figure S3). For the trees at MS through years without drought (2015−2017), AM2 yielded too steep an increase of SRD in early growing season and thus overestimated SRD through the corresponding time in most cases (e.g., Figure 4a,c, Supporting Information S1: Figure, S4). In drought years, AM2 resulted in a slightly better curve than without drought for one tree (Pentti; Figure 4d,e) but unrealistic sigmoid curves in the case of the other one (Sylvi; Supporting Information S1: Figure S4).

### 4.2 Sensitivities of Growth and Assimilation Rates to Water and Temperature

**Figure 6:**
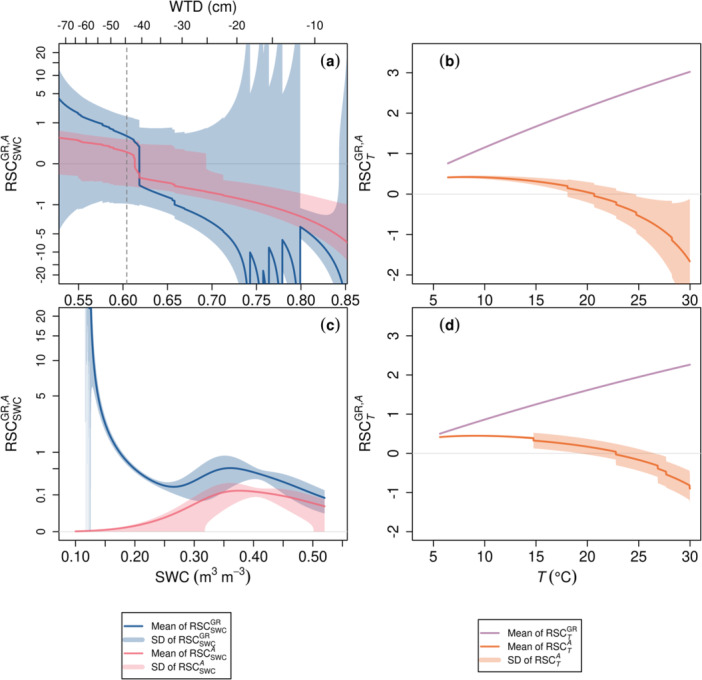
Relative sensitivity coefficients (RSC) of modelled growth rate (GR) and assimilation rate (*A*) to input variables soil water content (SWC, m^3^ m^−3^) and air temperature (T, °C) for the peatland (PS; a, b) and mineral‐soil (MS; c, d) sites. At PS, SWC and water table depth (WTD) are correlated (Equation 15), and the MAP estimate of optimal WTD (Table 2 and Supporting Information S1: Table S2) is displayed as dashed grey lines. The presented ranges of SWC and *T* are approximately those of observed values during the growing season. The means and the standard deviation (SD) intervals were calculated using 11 trees for PS and nine tree‐years for MS. SD of ${\mathrm{RSC}}_{T}^{\mathrm{GR}}$ (b, d) is minimal (on the scale of 10^−13^) and thus not displayed.

GR was more sensitive than assimilation rate (*A*) to SWC at both sites, whether or not waterlogging was present (Figure 6). The absolute value of GR‐to‐SWC RSC ($\left|{\text{RSC}}_{\text{SWC}}^{\text{GR}}\right|$) considerably increases at the dry and wet ends of SWC at PS and is almost always larger than $\left|{\text{RSC}}_{\text{SWC}}^{A}\right|$, although the variance between trees is large (Figure 6a). $\left|{\text{RSC}}_{\text{SWC}}^{\text{GR}}\right|$ is smaller at MS than at PS throughout the SWC range. The GR‐to‐temperature sensitivity (${\text{RSC}}_{T}^{\text{GR}}$) increases with increasing *T* in a quasi‐linear pattern similar at both sites, while ${\text{RSC}}_{T}^{A}$ declines due to increasing VPD with increasing *T* (Figure 6b,d). The sign of ${\text{RSC}}_{x}^{y}$ (Equation 25) is determined by δy/δx when *y* (e.g., *A*) and *x* (e.g., *T* in the current range) are always positive. Thus, the RSC results show that ∂A/∂T decreased with increasing *T* over *c*. 10−15°C, but the decrease was gradual and remained positive for Norway spruce in the peatland until *c*. 20°C (Figure 6b) and for Scots pine on mineral soils until 23°C (Figure 6d).

### 4.3 Correlation Between Growth Phenology and Photosynthetic Production

**Figure 7:**
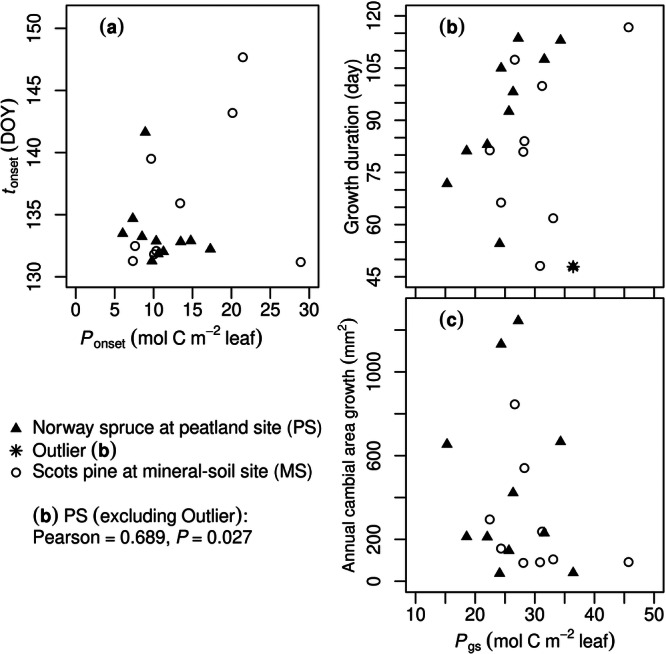
Growth and phenological traits in relation to assimilation rates. *P*_onset_ (a) and *P*_gs_ (b, c), leaf‐specific production summed over the period by the cambial growth onset and over the whole growing season, respectively; *t*_onset_ (a), timing of cambial growth onset. All Pearson's correlation coefficient's *p* > 0.1 except for one in (b), displayed on the bottom left.

The timing of cambial growth onset (*t*_onset_) and leaf‐specific production by the same time (*P*_onset_) were not significantly correlated for either site (Figure 7a), and neither were annual area growth and growing‐season leaf‐specific production (*P*_gs_; Figure 7c). Nevertheless, growth duration and *P*_gs_ were positively correlated for PS when an outlier was unused (Pearson's coefficient = 0.689, *p* = 0.027; but for MS *p *> 0.1; Figure 7b). The outlying sample tree (#3) at PS presented Cook's distance = 1.9 in the correlation analysis and also the lowest *R* ^2^ in SRD simulation (0.505; Supporting Information S1: Table S1a).

## 5 Discussion

We developed and parameterized a semi‐mechanistic model that couples stomatal control and cambial growth, and thus we were able to simulate growth, transpiration and assimilation rates simultaneously at a high temporal resolution of 30 min over whole growing seasons (Objective 1). Using the model, growth was found more sensitive than assimilation to temperature and soil water status (Objective 2), and photosynthetic production was not found to affect cambial growth onset or annual area increment despite the correlation between production and growth duration (Objective 3).

### 5.1 Model Structure

Model versions without the empirical Gompertz function for phenology (AM1) or using only the Gompertz function for growth (AM2) failed to reproduce the seasonal pattern of dendrometer observations, suggesting the necessity of both biophysical and phenological components of the growth model. Without the phenological control of active cambial cell number (Equation 23), AM1 yielded a quasi‐linear SRD during growth (e.g., Figure 2a). The linear growth pattern has been opposed by earlier observations (e.g., Wodzicki 1971), explained by the shortening of the enlargement phase as the growing season proceeds. Thus, SRD saturates at a ‘plateau’, and this saturation is accounted for in FM as enlargement is a prerequisite for cell division (Equations (22), (23), (24), (25), (26), Supporting Information S1: Method S3; Fritts, Shashkin, and Downes 1999; Vaganov, Hughes, and Shashkin 2006). This model structure results in fewer divisions in the later growing season. The improvement from AM1 to FM also supports the earlier conclusion that the Gompertz function is particularly good at simulating the number of enlarging cells (Cuny et al. 2013). Using the Gompertz function alone as in AM2, however, was not sufficient to simulate cambial growth accurately. AM2 generally overestimated the GR during early‐ to mid‐summer, notably on short (suppressed) trees (e.g., Figure 2b,d) and/or when annual cambial growth is low (e.g., Figure 4c,e). These failures support including biophysical limitations in the model, without which the constraints of unfavourable environmental conditions cannot be adequately addressed.

In contrast with Cowan‐Farquhar‐type OSM (Equation 1), another widespread definition of stomatal optimality is maximized assimilation. In this alternative method, the costs related to water conditions are not included in the objective function but formulated as constraints on maximizing photosynthesis. This allows for a more in‐depth description of whole‐tree water balance and its effects on stomatal conductance. Hölttä et al. (2017) employed this method and have shown that their model results are compatible with the Cowan−Farquhar method, provided that *λ* is adjusted as in Equation 8. Similar results have been confirmed for more general cases by Dewar et al. (2018). Wolf, Anderegg, and Pacala (2016), on the other hand, extended the Cowan‐Farquhar‐type OSM by expressing *E* explicitly as a function of leaf water potential. They defined the assimilation cost related to water potential as Θ to express

(30) $$\lambda ≝\frac{\partial A}{\partial E}=\frac{\partial \Theta }{\partial E}$$

under optimal stomatal behaviour. Mathematically, our expression of *λ* (Equation 8) can be interpreted as a solution to the Wolf et al.‐type optimization as such

(31) $$\Theta =10^{z_{0}}{\left(\frac{k_{\text{sl}}}{k_{0}}\right)}^{z_{1}}E$$

However, the correlation between *λ* and *k*_sl_ (Equation 8) that can be implemented into Cowan‐Farquhar‐type OSM was derived from the complex whole‐vasculature model by Hölttä et al. (2017) and was not found a priori. Nevertheless, the compatibility of this correlation with Wolf et al.‐type optimization (Equation 31) suggests that the parameters in Equations 8 and 31 embody a set of non‐stomatal processes and traits in photosynthesis, including intercellular CO_2_ kinetics, carboxylation capacity and CO_2_ compensation point (Hölttä et al. 2017; Dewar et al. 2018). Thus, using a numerically simpler formulation, the current model presents an attempt to integrate the aforementioned studies with different definitions of stomatal optimality.

### 5.2 Sensitivities of Growth and Photosynthesis to the Environment

The sensitivity analyses showed that GR at both sites was more sensitive than the assimilation rate to SWC and temperature (Objective 2; Figure 6), suggesting that effects of environmental factors on sink activities were stronger than those on source activities. Previous studies have reported such decoupled responses of growth and assimilation to soil water deficit (e.g., Hsiao 1973; Bogeat‐Triboulot et al. 2007; Eckes‐Shephard et al. 2021), which result in NSC accumulation in various plant parts instead of carbon starvation (Muller et al. 2011). The resilience of carbon gain to water stress may be related to the insensitivity of photosynthetic enzymes (e.g., Rubisco) to leaf water content (Flexas et al. 2006). Thus, assimilation may not immediately decline as much as SWC does, although carbon influx is indeed inhibited in the longer term if low stomatal conductance persists. In contrast, the turgor of cambial cells is sensitive to water deficit and instantly restrains their division rate under dry conditions (Equations (19), (20), (21), (22), (23), (24)). Therefore, assimilation and carbon availability can sustain over declining water potential, if not prolonged, before becoming the limiting factor of growth, especially in the current intra‐annual view. Similarly, the responses of growth and assimilation to temperature were also decoupled. The positive effect of *T* on GR is larger than *T* on *A*, and ∂A/∂T becomes negative at higher *T* than *c*. 20°C or *c*. 23°C (Norway spruce at PS and Scots pine at MS, respectively; Figure 6b,d) while ∂[GR]/∂T remains positive through 30°C. These results suggest the stronger activation of cambial‐growth‐related sink activities than assimilation by temperature and/or the limiting effects of increasing VPD with increasing temperature on photosynthesis via stomatal closure. The decoupling of environmental disturbances and assimilation may be related to the use of NSC storage under unfavourable conditions (Linkosalo, Hakkinen, and Hanninen 2006; Schiestl‐Aalto et al. 2015; Hartmann and Trumbore 2016), and thus carbon source limitation may occur on a larger temporal scale (e.g., annual to decadal) under prolonged impedance of NSC replenishment whereas sink limitation is crucial at the shorter term (monthly or intra‐annual).

The sensitivity analyses also imply on‐site‐ and/or species‐specific acclimation to environments. Growth of the Norway spruce at PS was more dependent on SWC than on *T*, whereas that of Scots pine at MS had such dependence only under very low SWC (< *c*. 0.18 m^3^ m^−3^). The higher tolerance to dry conditions of Scots pine at MS than Norway spruce at PS is also reflected by the difference in estimated *ψ*_0_ (threshold water potential for growth, −0.6 MPa for PS and −1.1 MPa for MS; Supporting Information S1: Tables S2, S3). This difference in tolerance may be related to the distinction between the species root system structure, that is, Norway spruce has the plate root system mainly spread to the shallow soils while the tap root system of Scots pine extends into deeper soils (Kalliokoski, Nygren, and Sievänen 2008). However, it should be noted that during the present sensitivity analyses, the other environmental factors were held constant, while in reality, correlations exist between them and, thus, the optimum of a factor may be affected. More specific studies on site‐ and/or species‐specific acclimation integrated with the current framework are needed for drawing more certain conclusions.

### 5.3 Cambial Growth Phenology Versus Carbon Gain

At the current sites, the trees' maximum assimilation rate was simulated to be 11.8 µmol C m^−2^ leaf s^−1^, a moderate level compared with typical boreal conifers (Hall et al. 2013; Crous, Uddling, and De Kauwe 2022), representing one of the least productive biomes of the globe (Cramer et al. 1999). When carbon gain is limited in early spring, growth may be downregulated and delayed (Huang et al. 2021), and thus a connection between growth phenology and carbon source was hypothesized (Objective 3). Although the phenological control is emphasized by comparing FM and AMs, its linkage to photosynthetic production is hardly supported by correlation analysis. No significant correlation was detected between phenological traits and leaf‐specific production at MS. At PS, the relations between phenological traits and leaf‐specific production varied as growing season proceeded, changing from decoupled (no significant correlation between *t*_onset_ and *P*_onset_; Figure 7a) to correlated (growth duration and *P*_gs_, excluding a statistical outlier; Figure 7b). Nonetheless, the annual cambial area increment was not correlated with *P*_gs_ at either site (Figure 7c). Still, the significant correlation at PS supports the role of carbon availability in determining growth duration under favourable water conditions (Schiestl‐Aalto et al. 2015; Cartenì et al. 2018; Cartenì et al. 2023), albeit carbon allocation or storage dynamics is not presented in the current model. The insignificant results, more generally, agree with the decoupling of phenology from GPP (Delpierre, Berveiller, et al. 2016) and of growth duration from growth itself (Camarero et al. 2022) found on temperate deciduous and boreal coniferous trees. Such decoupling may be related to the varying sensitivity of cell development phases to carbon availability, which impacts cell wall thickening more than the turgor‐driven cytoplastic expansion (Cuny et al. 2015; Friend, Eckes‐Shephard, and Tupker 2022). Growth phenology should encapsulate other factors and processes additional to carbon gain, for example, hormonal control (Vaganov, Hughes, and Shashkin 2006; Hänninen 2016; Hartmann et al. 2021), which results in a relatively consistent timing of growth onset that is less dependent on the current photosynthetic supply. Therefore, from a modelling perspective, phenology per se should be accounted as a regulator of growth‐related sink activities, and carbon gain is not sufficient to replace phenology for this regulation. This methodology should be suitable for modelling tree growth in other harsh environments featuring annual cycles that suppress both carbon source and sink activities (e.g., alpine), provided local calibrations of the model.

### 5.4 Potential Improvements

Despite the overall good performance of the model, it could be further enhanced as follows. The SRD simulation of trees in drained peatland was better than in mineral soils, and significant droughts (e.g., in 2018) exacerbated the model performance on SRD at MS, especially following rain events (Supporting Information S1; Table S1, Figures 4d and 5h). The model may be enhanced with this respect by including variable MOE dependent on water potential (De Schepper and Steppe 2010) or on growth progress. Also, introducing other water storages (e.g., parenchyma) into the model and improving the quantification of their dynamics may also enhance SRD prediction upon rain events. These potential improvements should increase model complexity and require more demanding measurements and observations for testing, for example, chrono‐sequences of water potential and soil and rhizospheric structures deeper than the B horizon. Despite such difficulties, tree growth responding to drought and sudden rain should be scrutinized using experimental and modelling methods as extreme weather events (including heavy precipitation and droughts) have likely become more frequent and intense globally under the changing climate (Seneviratne et al. 2021). Furthermore, hormonal regulation, which is the controlling mechanism of wood formation phenology (e.g., Buttò et al. 2020), may also be integrated into the framework. Previous modelling studies have shown the algebraic resemblance of the diffusion of growth‐related hormones to the Gompertz function (Hartmann et al. 2021) as well as the method of proxying hormonal regulation using NSC dynamics (Cartenì et al. 2018; Friend, Eckes‐Shephard, and Tupker 2022). This enhancement should improve our understanding of growth mechanisms especially in the context of source versus sink limitations.

Additionally, cambial growth dynamics are not necessarily the same as whole‐tree growth dynamics, and at the whole‐tree level carbon balance should be addressed. Therefore, growths of other tree compartments (e.g., foliage) and dynamics of carbon allocation and storage should be incorporated into the model for elaborating source versus sink limitations on whole‐tree growth and its connection to phenology. Accounting for carbon storage should also provide insights into carbon source versus sink pathways of environmental control, a key issue in growth and DVM (Fatichi et al. 2019; Friend et al. 2019; Friend, Eckes‐Shephard, and Tupker 2022). This enhancement may be realized by bridging the current model with another one based on carbon analysis and phenology (e.g., Schiestl‐Aalto et al. 2015) via photosynthesis and cambial growth.

## 6 Conclusions

The biophysical formulation of sink activities and empirical description of growth phenology are both indispensable for modelling intra‐annual cambial growth of boreal trees. On fine temporal scales, growth is more sensitive than assimilation to temperature and soil water, and, thus, carbon source plays a minor role. Carbon gain affected growth duration in peatland but did not affect other phenological traits (growth onset or annual cambial increment) in either peatland or mineral soil. Hence, the phenological regulation of growth is hardly due to carbon gain, and phenology per se should be explicitly expressed in cambial growth modelling. Our study provides a modelling framework for clarifying the effects of sink and carbon source activities and phenology on cambial growth as well as an example of using Bayesian inference for parameterizing complex eco‐physiological models efficiently. Important further research includes (1) elaborating how the parameters of the Gompertz function vary with environmental conditions, which is important for (2) developing a mechanistic description of growth phenology incorporating NSC allocation and/or hormonal regulation and (3) integrating the current model with dynamic vegetation models for more accurate and robust predictions about the changing forests in the future.

## Conflicts of Interest

The authors declare no conflicts of interest.

## Supporting information

Supporting information.

Supporting information.

Supporting information.

Supporting information.

Supporting information.

## Data Availability

The data that support the findings of this study are openly available in Zenodo at https://zenodo.org/, reference number 10037224, 10043607.
