# Supplementary figures and images for: A Coupled Model of Hydraulic Eco‐Physiology and Cambial Growth — Accounting for Biophysical Limitations and Phenology Improves Stem Diameter Prediction at High Temporal Resolution
Source: Plant Cell Environ. 2024 Oct 24;48(2):1344–65. doi: 10.1111/pce.15239 (PMC11695789; doi:10.1111/pce.15239)

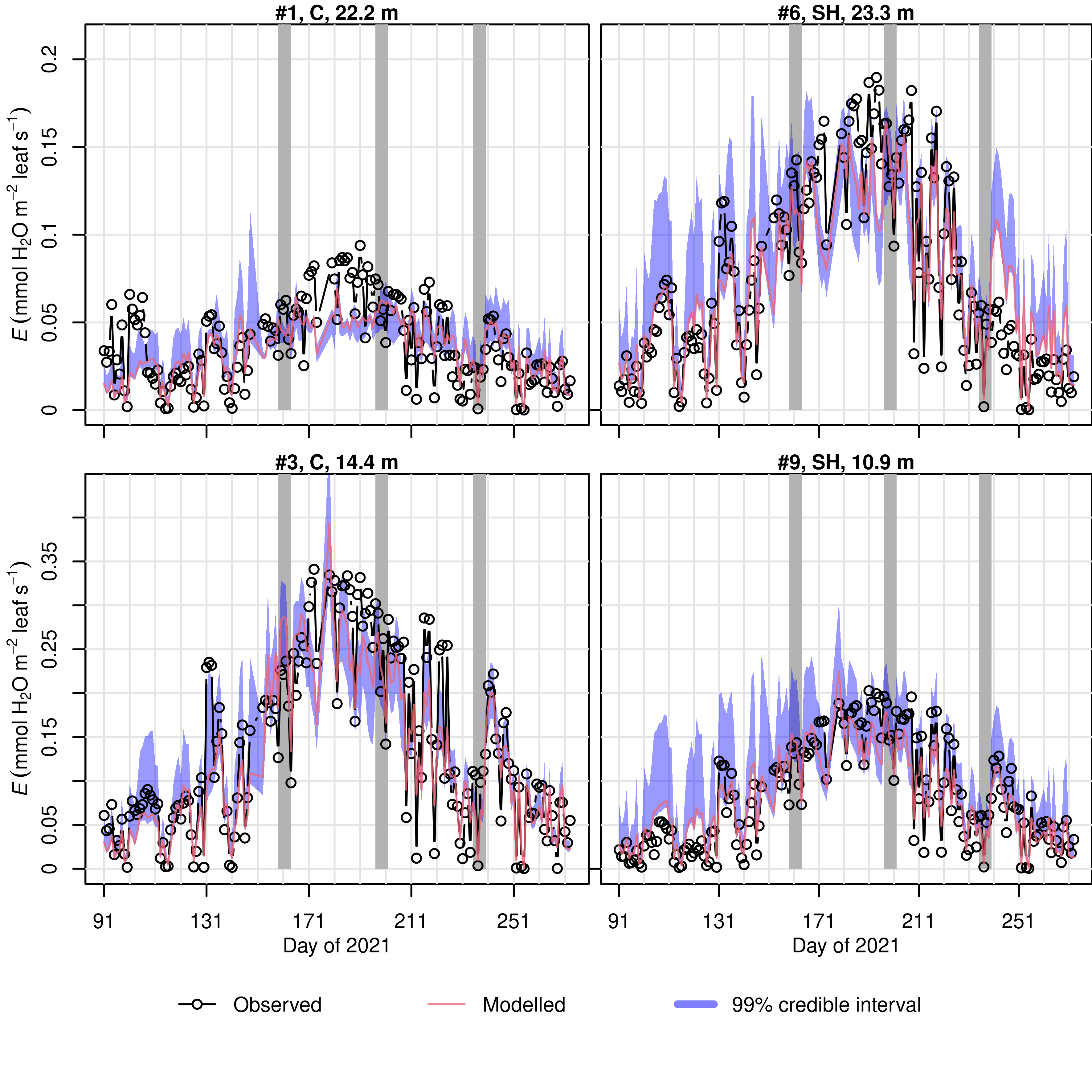

Supplement: Supplementary file 1 — Supporting information. [file PCE-48-1344-s005.tiff]

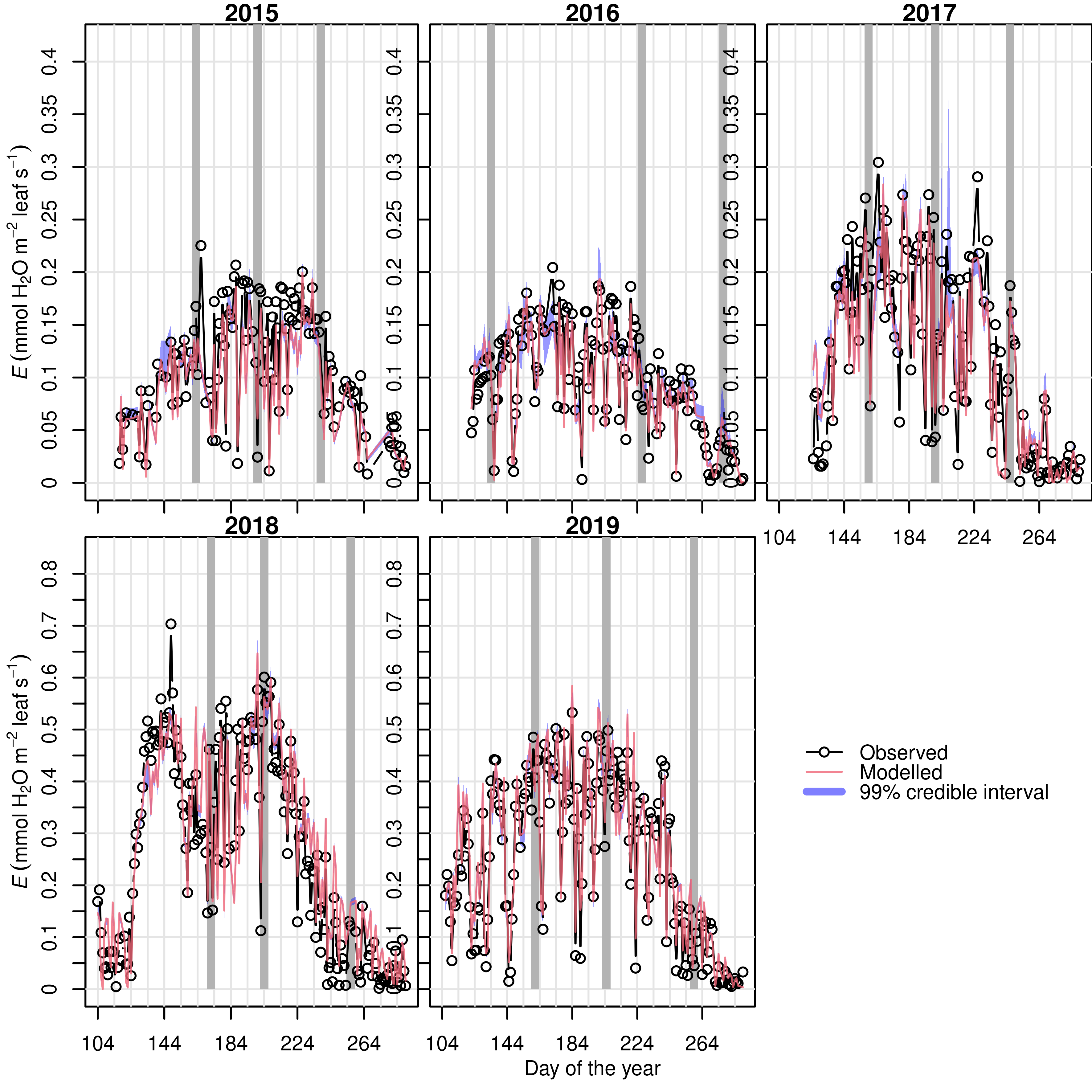

Supplement: Supplementary file 2 — Supporting information. [file PCE-48-1344-s001.tiff]

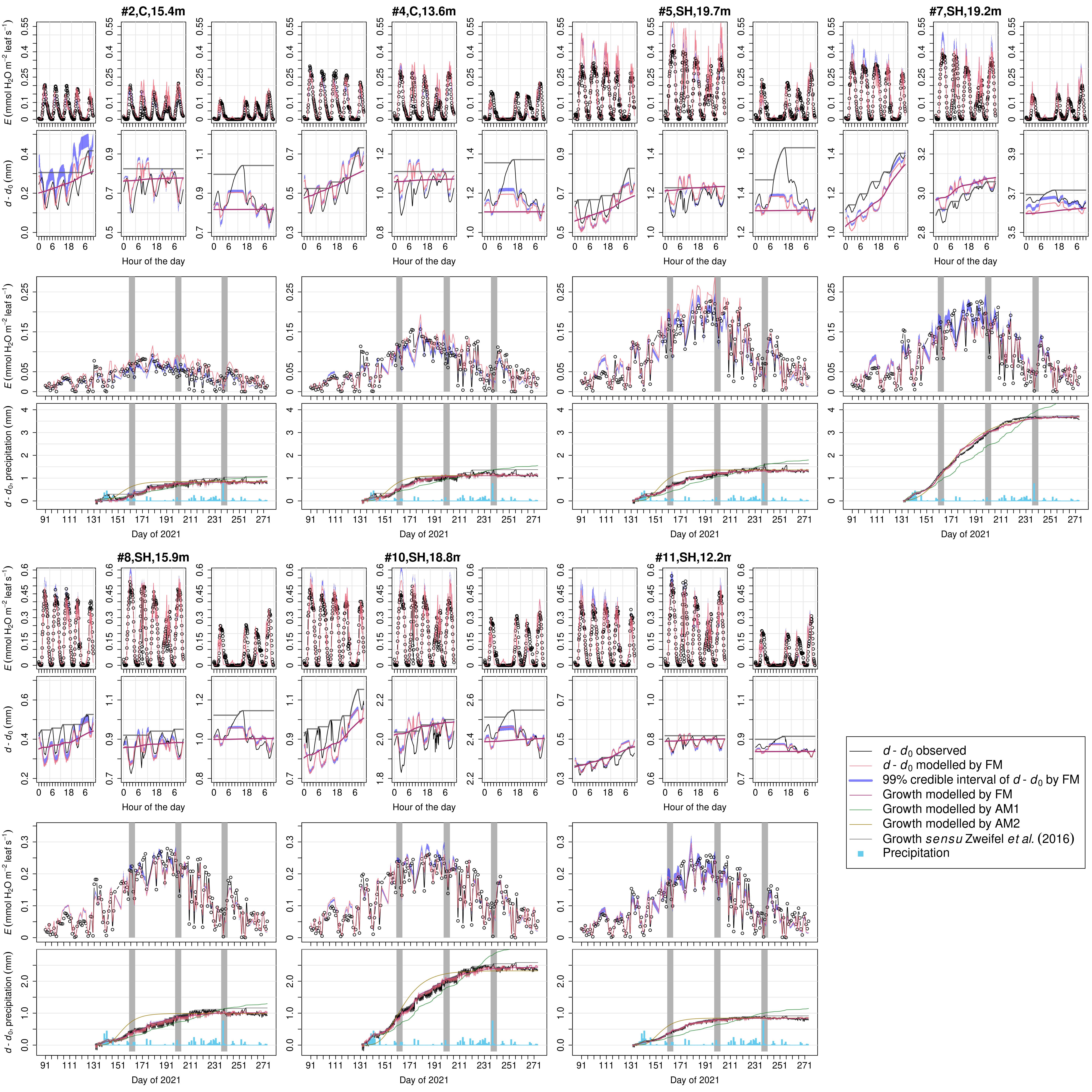

Supplement: Supplementary file 3 — Supporting information. [file PCE-48-1344-s003.tiff]

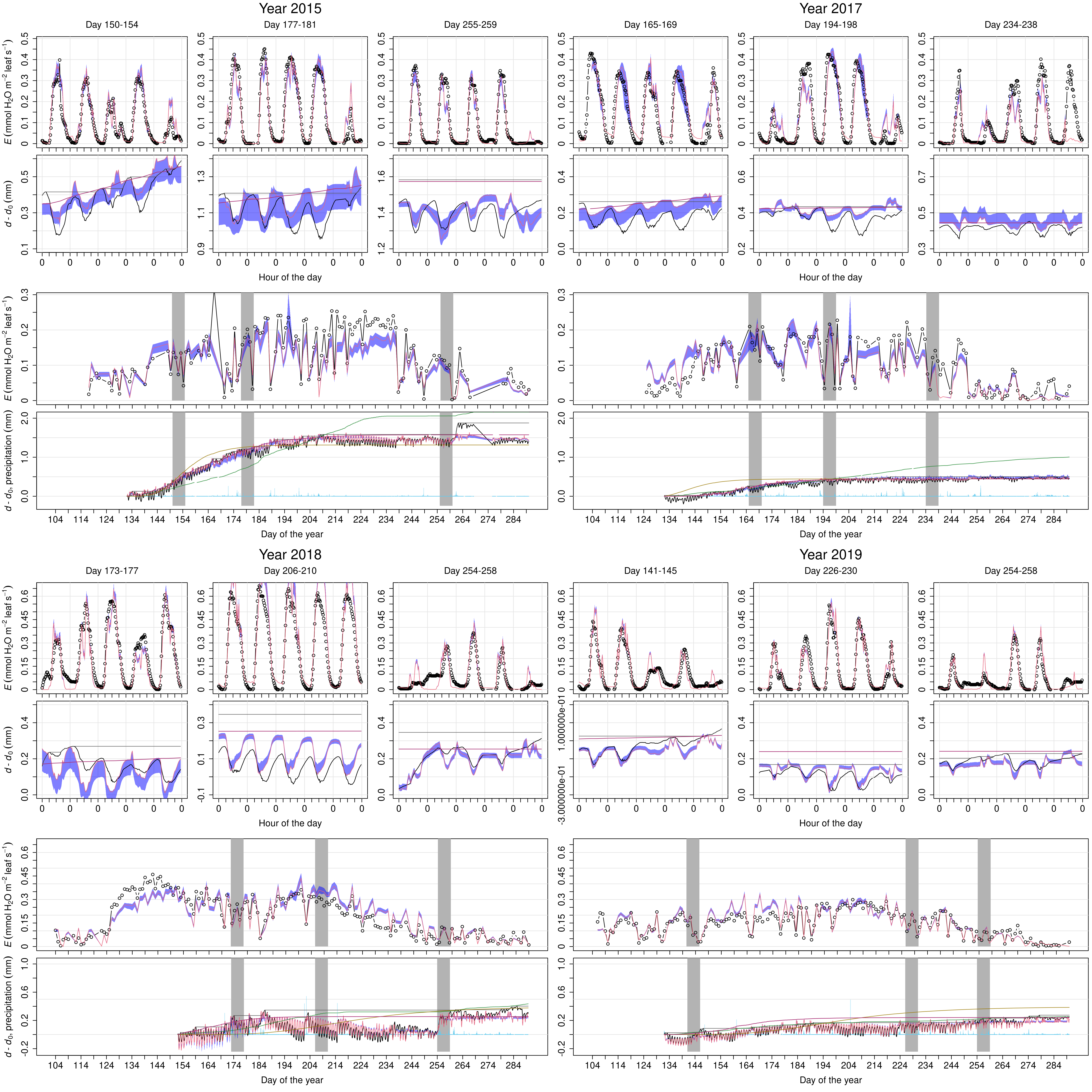

Supplement: Supplementary file 4 — Supporting information. [file PCE-48-1344-s004.tiff]
