# Supplementary material for: A Coupled Model of Hydraulic Eco‐Physiology and Cambial Growth — Accounting for Biophysical Limitations and Phenology Improves Stem Diameter Prediction at High Temporal Resolution
Source: Plant Cell Environ. 2024 Oct 24;48(2):1344–65. doi: 10.1111/pce.15239 (PMC11695789; doi:10.1111/pce.15239)
Supplement: Supplementary file 5 — Supporting information. [file PCE-48-1344-s002.docx]

**Supplementary Materials**

Article title: A coupled model of hydraulic eco-physiology and cambial growth – accounting for biophysical limitations and phenology improves stem diameter prediction at high temporal resolution

Authors: Che Liu, Mikko Peltoniemi, Pavel Alekseychik, Annikki Mäkelä, and Teemu Hölttä

The following Supporting Information is available for this article:

**Methods S1** Formulation of soil-to-tree base conductance (*k*_sb_)

**Methods S2** Analysis of the bark as a linear hydraulic system with rainwater effects

**Methods S3** The linear relative expansion rate of cambial cells

**Methods S4** Data model for parameter estimation

**Note S1** On the turgor-independent modulus of elasticity (MOE)

**Note S2** On the temperature effects in modelling cambial growth

**Table S1** Statistics of model performance on individual trees

**Table S2** Maxima a posteriori (MAP) estimates and 99% intervals of the posterior distributions of parameters for Ränskälänkorpi

**Table S3** MAP estimates and 99% intervals of the posterior distributions of parameters for Hyytiälä

**Fig. S1** Model results of transpiration rate (*E*) of the other Norway spruce trees in Fig. 1 & 2

**Fig. S2** Model results of *E* of the Scots pine tree (named Pentti) in Fig. 3 & 4

**Fig. S3** Model results of the other Norway spruce trees at Ränskälänkorpi

**Fig. S4** Model results of the other Scots pine tree (named Sylvi) at Hyytiälä.

**Methods S1** Formulation of soil-to-tree base conductance (*k*_sb_)

The formulation of *k*_sb_ follows the analysis by Nikinmaa *et al.* (2014). The Hagen-Poiseuille equation shows that hydraulic conductivity (*K*) is proportional to the number (*n*) and the fourth power of radius (*r*) of the conduits ($K\propto{nr}^{4}$). On the whole-tree scale, the tapering of *r* has been modelled as $r\propto l^{1/4}$ (*l*, water transport distance; West *et al.*, 1999). The total conducting area through the stem has been found approximately constant ($nr^{2}=\mathrm{constant}$; Savage *et al.*, 2010), and thus the conduit number (per cross-sectional area of stem) is negatively correlated with the second power of *r* ($n\propto r^{-2}$). Combining these equations gives $K\propto{r^{-2}r}^{4}=r^{2}\propto l^{1/2}\Rightarrow K\propto l^{1/2}$. This consequent correlation leads to the expression of relative root-to-tree base (*K*_rb_) to root-to-leaf (*K*_rl_) conductivities as

|  | $\frac{K_{\mathrm{rb}}}{K_{\mathrm{rl}}}=\left( \frac{l_{\mathrm{rb}}}{l_{\mathrm{rl}}} \right)^{\frac{1}{2}}$ | Eqn S1.1 |
| --- | --- | --- |

where in the current study *l*_rb_ and *l*_rl_ were assumed to be the heights from roots (depth 0.2 m) to breast height and to the treetop, respectively (Table 1), and thus they can be calculated using tree height (Table 3).

Hydraulic conductance (*k*) is defined as the differentiated *K* with respect to *l* ($k≝{dK}/{dl}$). Hence, differentiating Eqn S1.1 results in

|  | $\frac{k_{\mathrm{rb}}}{k_{\mathrm{rl}}}=\left( \frac{l_{\mathrm{rb}}}{l_{\mathrm{rl}}} \right)^{-\frac{1}{2}}$ | Eqn S1.2 |
| --- | --- | --- |

where *k*_rl_ was estimated using Eqn 16. Use *k*_rb_ resulting from Eqn S2.2 and *k*_sb_ can be calculated as a series of conductances similar to Eqn 8, i.e.

|  | $k_{\mathrm{sb}}^{-1}=k_{\mathrm{sr}}^{-1}+k_{\mathrm{rb}}^{-1}$ | Eqn S1.3 |
| --- | --- | --- |

where *k*_sr_ was modelled by Eqn 14 (drained peatland) or 12 (mineral soils).

**References for Method S1**

Nikinmaa E, Sievänen R, Hölttä T. 2014. Dynamics of leaf gas exchange, xylem and phloem transport, water potential and carbohydrate concentration in a realistic 3-D model tree crown. *Annals of botany* 114(4): 653-666

Savage VM, Bentley LP, Enquist BJ, Sperry JS, Smith DD, Reich PB, von Allmen EI. 2010. Hydraulic trade-offs and space filling enable better predictions of vascular structure and function in plants. *Proceedings of the National Academy of Sciences of the USA* 107: 22722–22727

West GB, Brown JH, Enquist BJ. 1999. A general model for the structure and allometry of plant vascular systems. *Nature* 400: 664–667.

**Methods S2** Analysis of the bark as a linear hydraulic system with rainwater effects

As continuous measurements of water content in the bark (*q*_b_) or potential difference between bark and cambium ($\Delta\psi_{\mathrm{bc}}$) were not available for us, we modelled them for better capturing the dynamics of stem radial dimension during and after rain events (Eqns 20 and 21, Fig. 1b,c). The bark was modelled as a linear hydraulic system and thus features a time constant (*τ*_b_). Hence, the dynamics of *q*_b_ follow

|  | $\dot{q}_{b}\left( t \right)=J_{\mathrm{bin}}\left( t \right)-J_{\mathrm{bc}}\left( t \right)=J_{\mathrm{bin}}\left( t \right)-\frac{q_{b}\left( t \right)}{\tau_{b}}$ | Eqn S2.1 |
| --- | --- | --- |

($q_{b}\left( 0 \right)=0$ at the initial state) where *J*_bin_ and *J*_bc_ are, respectively, water fluxes input to the bark and output from the bark to the cambium. As we were not aware of any direct measurement of its value during natural rain events, we evaluated *J*_bin_ using rain intensity (*w*_r_, mm s^-1^) directly until it reaches a maximum value ($J_{\mathrm{bin}}^{\max}$, measured by Gimeno *et al.* [2022] experimentally; Table 1). That is to say

|  | $J_{\mathrm{bin}}\left( t \right)=min\left\{ w_{r}\left( t \right),J_{\mathrm{bin}}^{\max} \right\}$ | Eqn S2.2 |
| --- | --- | --- |

Considering the bark model’s analogy to the RC (resistor-capacitor) circuit in electricity, its time constant is

|  | $\tau_{b}=\frac{C_{b}}{k_{\mathrm{bc}}}$ | Eqn S2.3 |
| --- | --- | --- |

where *C*_b_ is the hydraulic capacitance of the bark, and *k*_bc_ the hydraulic conductance between bark and cambium. The definitions of linear modulus of elasticity ($\mathcal{E≝}{\partial\psi}/\left( {\partial d}/{d_{0}} \right)$) and hydraulic capacitance ($C≝{\partial q}/{\partial\psi}$) can be combined if 1) *q* is expressed as mm^3^ water mm^-2^ bark = mm and thus equivalent to *d*, and 2) osmotic and gravitational potentials are assumed constant (same assumptions as for Eqn 19 in the main text), i.e. $\partial\psi=\partial\psi_{p}+\partial\psi_{\pi}+\partial\psi_{g}$ with $\partial\psi_{\pi}=0, \partial\psi_{g}=0$. Hence,

|  | $C_{b}=\frac{d_{b0}}{\mathcal{E}_{b}}$ | Eqn S2.4 |
| --- | --- | --- |

and thus Eqn S1.3 becomes

|  | $\tau_{b}=\frac{d_{b0}}{k_{\mathrm{bc}}\mathcal{E}_{b}}$ | Eqn S2.5 |
| --- | --- | --- |

where *d*_b0_ (the initial bark thickness) can be measured or estimated, *k*_bx_ and $\mathcal{E}_{b}$ estimates available from previous studies (Table 1). Hence,

|  | ${\Delta\psi}_{\mathrm{bc}}\left( t \right)=\frac{J_{\mathrm{bc}}\left( t \right)}{k_{\mathrm{bc}}}=\frac{q_{b}\left( t \right)}{k_{\mathrm{bc}}\tau_{b}}=q_{b}\left( t \right)\frac{\mathcal{E}_{b}}{d_{b0}}$ | Eqn S2.6 |
| --- | --- | --- |

and thus,

|  | $\frac{d\left( {\Delta\psi}_{\mathrm{bc}} \right)}{dt}=\frac{\dot{q}_{b}\left( t \right)}{C_{b}}=\dot{q}_{b}\left( t \right)\frac{\mathcal{E}_{b}}{d_{b0}}$ | Eqn S2.7 |
| --- | --- | --- |

where $\dot{q}_{b}$ can be calculated using Eqns S2.1, S2.2 and S2.5 combined. Eqn S2.7 is used in Eqns 20 and 21.

The bark-to-xylem flow was not explicitly expressed in the stomatal model for modelling transpiration rate (*E*) for the following reasons. First, water potentials of xylem and cambium were consistently assumed to be equilibrated instantly (see Section 2.2 of the main text including Fig. 1b,c). Second, on the annual time span of the current data (cf. several days as in the experiment by Gimeno *et al.* [2022]), the total *q*_b_ (per *bark* area) to total sap flow (per *sapwood* area) ratio of a year is small, ranging from 1.03% to 5.38% at Ränskälänkorpi and from 0.55% to 3.10% at Hyytiälä. Moreover, introducing the bark-to-xylem flow into modelling *E* would require unifying the area base of *q*_b_ and sap flow density and thus bark-to-sapwood areas ratio. However, the estimation of this ratio should introduce considerable error due to the heterogeneous tapering of tree trunks. Also, the simplified model of water uptake through the bark (e.g. Eqn S2.2) may have induced structural errors, which yet requires more quantitative experimental measurements to clarify.

**Reference for Method S2**

Gimeno TE, Stangl ZR, Barbeta A, Saavedra N, Wingate L, Devert N, Marshall JD. 2022. Water taken up through the bark is detected in the transpiration stream in intact upper‐canopy branches. *Plant, Cell & Environment* 45(11): 3219-3232.

**Methods S3** The linear relative expansion rate of cambial cells in comparison with Cabon *et al.* (2020)

Cabon *et al.* (2020) employed the *volumetric* relative expansion rate of cambial cells (*p*_V_; NB the change of notation in the current study) for modelling growth, and defined it as

|  | $p_{V}\left( t \right)=\frac{\dot{V}}{V}$ | Eqn S3.1 |
| --- | --- | --- |

where *V* is cell volume. By using Eqn S3.1 and assuming that volume doubling is the prerequisite for splitting, Cabon *et al.* (2020) have demonstrated that the total occurrences of cell splitting (*N*_D_) by a time *t_x_* is

|  | $N_{D}=\log_{2} \frac{V\left( t_{x} \right)}{V\left( 0 \right)}=\frac{\ln\frac{V\left( t_{x} \right)}{V\left( 0 \right)}}{\ln2}=\frac{1}{\ln2}\int_{0}^{t_{x}} \frac{\dot{V}}{V}dt=\frac{1}{\ln2}\int_{0}^{t_{x}} p_{V}\left( t \right)dt$ | Eqn S3.2 |
| --- | --- | --- |

and thus its rate is

|  | $\dot{N}_{D}=\frac{p_{V}\left( t \right)}{\ln2}$ | Eqn S3.3 |
| --- | --- | --- |

which would render Eqn 17 as

|  | $\dot{d}_{\mathrm{gro}}=N_{c}\dot{N}_{D}\bar{V}=N_{c}\frac{p_{V}}{\ln2}\bar{V}$ | Eqn S3.4 |
| --- | --- | --- |

However, in the current model for the consistency with other modelled variables (e.g. $\dot{d}_{\mathrm{hyd}}$, Eqn 13), the *linear* (radial) relative expansion rate (*p*) is used, which is

|  | $p=\frac{\dot{d}_{c}}{d_{c}}=\frac{1}{3}p_{V}$ | Eqn S3.5 |
| --- | --- | --- |

(*d*_c_, cell radial diameter) provided that the expansion rate equals on all the three dimensions. Correspondingly, $\phi_{i,a}^{\max}$ took the prior range for calibration (Table 2) based on Cabon *et al.* (2020) results divided by 3 (additional to converting d^-1^ to h^-1^).

**Reference for Method S3**

Cabon A, Peters RL, Fonti P, Martínez‐Vilalta J, De Cáceres M. 2020. Temperature and water potential co‐limit stem cambial activity along a steep elevational gradient. *New Phytologist* 226(5): 1325-1340.

**Methods S4** Data model for parameter estimation

The data model includes two probability density functions (PDFs) for the errors of OSM and the SRD model, respectively. The error matrix of OSM was yielded by comparing *J*^(O)^/*ρ* and modelled transpiration rate (*E*^(M)^; Eqn 3). The time lag between *J*^(O)^ and *E*^(M)^, mainly due to trees’ water storage, was introduced into the error calculation as a dependent of tree height (*h*; Table 3) with a linear slope (*β*; Liu *et al.*, 2020), which was estimated along with the other parameters. *J*^(O)^ was interpolated between each two data points for a smoother random walk for parameterization. Hence, for tree *i* at any time point *t* between data points *t*_1_ and *t*_2_ within the study period, the error of OSM is

|  | $\varepsilon_{i}^{O}\left( t \right)=\frac{\left\vert\beta h_{i}-t_{2} \right\vert J_{i}^{(O)}\left( t+t_{1} \right)+\left\vert\beta h_{i}-t_{1} \right\vert J_{i}^{(O)}\left( t+t_{2} \right)}{\rho_{i}\left\vert t_{2}-t_{1} \right\vert}-E_{i}^{(M)}\left( t \right)$ | Eqn S4.1 |
| --- | --- | --- |

where *ρ* is leaf-to-sapwood areas ratio (Table 3). As $g_{0}$ truncates the continuity of $g_{\sigma}^{*}$ (Eqn 2) at near-zero values and correspondingly causes high probabilities of minimal *ε*^O^, PDF of the Laplace (cf. Gaussian) distribution was applied to describing the probabilities of OSM error ($℘_{O}$) with linear heteroscedasticity, that is,

|  | $℘_{O}\left( \varepsilon_{i}^{O}\left( t \right) \right)=\frac{1}{2\left( \alpha^{O}+\beta_{i}^{O}\varepsilon_{i}^{O}\left( t \right) \right)}\exp\left( -\frac{\left\vert\varepsilon_{i}^{O}\left( t \right) \right\vert}{\alpha^{O}+\beta_{i}^{O}E_{i}^{(M)}\left( t \right)} \right)$ | Eqn S4.2 |
| --- | --- | --- |

where *α*^O^ and *β*^O^ are intercept and slope parameters of the PDF’s rate, respectively. The other PDF in the data model describes the probability distribution of the error between observed (*d*^(O)^) and modelled SRD ($d^{(M)}\left( t_{x} \right)=\int_{0}^{t_{x}} \dot{d}^{(M)}\left( t \right)dt$, where *t_x_* is any time point within the woody growth period, and $\dot{d}^{(M)}\left( t \right)$ as $\dot{d}$ in Eqn 12), i.e.

|  | $\varepsilon_{i}^{R}\left( t \right)=d_{i}^{(O)}\left( t \right)-d_{i}^{(M)}\left( t \right)$ | Eqn S4.3 |
| --- | --- | --- |

Its PDF is the Gaussian distribution with linear heteroscedasticity, i.e.

|  | $℘_{R}\left( \varepsilon_{i}^{R}\left( t \right) \right)\mathcal{=N}\left( 0,\alpha^{R}+\beta_{i}^{R}d_{i}^{(M)}\left( t \right) \right)$ | Eqn S4.4 |
| --- | --- | --- |

where *α*^R^ and *β*^R^ are intercept and slope parameters. The total error likelihood of a site ($℘_{\mathrm{tot}}$) is the product of all the probabilities of ***ε***^O^ and ***ε***^R^ over the respective study periods ***t***^O^ and ***t***^R^ of *N* sample trees, i.e.

|  | $℘_{\mathrm{tot}}=\prod_{i=1}^{N} \left[ \prod_{t\in\boldsymbol{t}^{O}} ℘_{O}\left( \varepsilon_{i}^{O}\left( t \right) \right)\cdot\prod_{t\in\boldsymbol{t}^{R}} ℘_{R}\left( \varepsilon_{i}^{R}\left( t \right) \right) \right]$ | Eqn S4.5 |
| --- | --- | --- |

All estimated parameters were assumed independently and uniformly distributed *a priori* (Table 2).

**Reference for Method S4**

Liu Ch, Hölttä T, Tian X, Berninger F, Mäkelä A. 2020. Weaker light response, lower stomatal conductance and structural changes in old Boreal conifers implied by a Bayesian hierarchical model. *Frontiers in Plant Science* 11: 579319.

**Note S1** On the turgor-independent modulus of elasticity (MOE)

When modelling the elastic changes of stem radial dimension (SRD; Eqn 16), we assumed the tree-year-specific modulus of elasticity (MOE, $\mathcal{E}$) independent of turgor i.e. constant for one tree-year, same as in earlier studies (e.g. Hölttä *et al.*, 2010; Chan *et al.*, 2016; Mencuccini *et al.*, 2017). Indeed, on a wide range of turgor (0 to -3 MPa or even lower) the turgor-SRD correlation (*P*-*V* curve) resembles the logistic curve, and thus MOE is dependent on turgor (e.g. Tyree & Yang, 1990; Jones, 2014; Dietrich *et al.*, 2018). Nevertheless, on our narrower range of turgor (0 to no lower than -1.5 MPa) the *P-V* correlation is approximately linear, and thus it is acceptable to assume MOE constant over a variable turgor. Also, our narrow range of turgor might have caused the failure of our attempt to implement the logistic MOE-turgor correlation following De Scheppe & Steppe (2010). In this attempt, we could not obtain reasonable estimates of the coefficients of the logistic curve using the current data. Thus, for these reasons, we designed the MOE parameter as turgor-independent.

**References for Note S1**

Chan T, Hölttä T, Berninger F, Mäkinen H, Nöjd P, Mencuccini M, Nikinmaa E. 2016. Separating water‐potential induced swelling and shrinking from measured radial stem variations reveals a cambial growth and osmotic concentration signal. *Plant, Cell & Environment* 39(2): 233-244.

De Schepper V, Steppe K. 2010. Development and verification of a water and sugar transport model using measured stem diameter variations. *Journal of Experimental Botany* 61(8): 2083-2099.

Dietrich L, Zweifel R, Kahmen A. 2018. Daily stem diameter variations can predict the canopy water status of mature temperate trees. *Tree Physiology* 38(7): 941-952.

Hölttä T, Mäkinen H, Nöjd P, Mäkelä A, Nikinmaa E. 2010. A physiological model of softwood cambial growth. *Tree Physiology* 30(10): 1235-1252.

Jones HG. 2014. *Plants and Microclimate: A Quantitative Approach to Environmental Plant Physiology* (3^rd^ ed.). New York, NY, USA: Cambridge University Press.

Mencuccini M, Salmon Y, Mitchell P, Hölttä T, Choat B, Meir P, O'grady A, Tissue D, Zweifel R, Sevanto S. 2017. An empirical method that separates irreversible stem radial growth from bark water content changes in trees: theory and case studies. *Plant, Cell & Environment* 40(2): 290-303.

Tyree MT, Yang Sh-D. 1990. Water-storage capacity of *Thuja*, *Tsuga* and *Acer* stems measured by dehydration isotherms: the contribution of capillary water and cavitation. *Planta* 182: 420-426.

**Note S2** On the temperature effects in modelling cambial growth

It should be noted that no duplicate effects are expressed in modelling cambial growth, albeit both the Lockhart equation (Eqn 24) and the enzymatic thermodynamic properties (Δ*Η*_A_, Δ*Η*_D_ and Δ*S*_D_; Eqn 25) are present. The thermodynamic parameters in Eqn 25 indeed reflect the activation of the enzymatic system related to cambial growth, but the parameters themselves are independent of temperature. The cell extensibility (*ϕ*) from Eqn 25, dependent on temperature in a bell-shaped curve (Cabon *et al.*, 2020), is used in Eqn 24 as the slope of the Lockhart equation, and there is no temperature effect accounted for in Eqn 24 otherwise. Therefore, the only direct expression of temperature effects on growth is the structure of Eqn 25.

**Reference for Note S2**

Cabon A, Peters RL, Fonti P, Martínez‐Vilalta J, De Cáceres M. 2020. Temperature and water potential co‐limit stem cambial activity along a steep elevational gradient. *New Phytologist* 226(5): 1325-1340.

**Table S1** Model performance on individual trees of **(a)** Norway spruce at Ränskälänkorpi (drained peatland, PS) and **(b)** Scots pine at Hyytiälä (mineral soil, MS).

**(a)**

|  | *E* | | | | *d* | | | |
| --- | --- | --- | --- | --- | --- | --- | --- | --- |
| Tree | FS | Int | *R*^2^ | RMSE | FS | Int | *R*^2^ | RMSE |
| 1 | 0.968 | 2.85 | 0.570 | 30.7 | 0.990 | 12.72 | 0.990 | 46.7 |
| 2 | 0.706 | 3.63 | 0.546 | 38.3 | 0.948 | 40.62 | 0.955 | 66.8 |
| 3 | 1.055 | 8.29 | 0.762 | 90.7 | 0.846 | 3.85 | 0.505 | 59.2 |
| 4 | 0.880 | 6.82 | 0.680 | 47.9 | 0.999 | ns | 0.972 | 63.7 |
| 5 | 0.703 | 24.1 | 0.779 | 68.8 | 1.016 | -15.61 | 0.985 | 52.6 |
| 6 | 0.881 | 11.9 | 0.675 | 52.7 | 0.994 | 19.31 | 0.993 | 74.5 |
| 7 | 0.901 | 7.89 | 0.764 | 54.5 | 1.006 | -15.89 | 0.995 | 88.2 |
| 8 | 0.960 | 9.34 | 0.811 | 64.8 | 1.003 | -3.32 | 0.977 | 52.5 |
| 9 | 0.930 | 9.87 | 0.693 | 60.0 | 0.976 | -3.45 | 0.869 | 31.6 |
| 10 | 0.887 | 17.6 | 0.808 | 65.9 | 1.010 | -18.51 | 0.993 | 67.1 |
| 11 | 0.935 | 12.4 | 0.772 | 65.0 | 0.996 | 1.99 | 0.993 | 23.3 |

**(b)**

|  | *E* | | | | *d* | | | |
| --- | --- | --- | --- | --- | --- | --- | --- | --- |
| Year | FS | Int | *R*^2^ | RMSE | FS | Int | *R*^2^ | RMSE |
| Tree ‘Pentti’ | | | | | | | | |
| 2015 | 0.981 | 15.2 | 0.828 | 46.8 | 0.990 | -29.72 | 0.968 | 63.8 |
| 2016 | 0.995 | 4.64 | 0.859 | 40.2 | 1.045 | -47.24 | 0.952 | 188.1 |
| 2017 | 0.965 | 7.03 | 0.799 | 67.6 | 0.966 | -20 | 0.980 | 39.7 |
| 2018 | 0.968 | -3.54 | 0.864 | 123.4 | 1.021 | 2.71 | 0.776 | 60.3 |
| 2019 | 0.941 | 4.26 | 0.892 | 96.1 | 1.264 | -17.28 | 0.896 | 43.6 |
| Tree ‘Sylvi’ | | | | | | | | |
| 2015 | 1.074 | 9.63 | 0.822 | 61.9 | 1.019 | -54.65 | 0.967 | 94.6 |
| 2017 | 0.944 | 10.06 | 0.749 | 60.2 | 1.076 | -54.68 | 0.950 | 49.8 |
| 2018 | 0.923 | 15.33 | 0.812 | 98.7 | 0.995 | 7.02 | 0.723 | 69.2 |
| 2019 | 0.829 | 19.87 | 0.845 | 68.3 | 0.943 | 7.01 | 0.832 | 37.0 |

*E*, transpiration rate; *d*, stem radial dimension; FS and Int, fitted slope and intercept of observed to modelled variables, respectively; RMSE, root-mean-square error. Both Int and RMSE are in µmol H_2_O m^-2^ leaf s^-1^ for *E* and µm for *d*. In **(b)**, data of Sylvi in 2016 were unavailable due to technical failure. All FS’s and Int’s *P* < 0.001, except for ns (non-significant, *P* > 0.05) and the values in grey shade (0.001 < *P* < 0.05).

**Table S2** Maxima a posteriori (MAP) estimates and 99% intervals of the posterior distributions of parameters for site Ränskälänkorpi

|  | MAP | 0.5% | 99.5% |  | MAP | 0.5% | 99.5% |
| --- | --- | --- | --- | --- | --- | --- | --- |
| *ξ*_m1_ | 0.199 | 0.191 | 0.236 | $\phi_{1}^{\max}$ | 15.0 × 10^-3^ | 13.2 × 10^-3^ | 16.8 × 10^-3^ |
| *ξ*_m2_ | 0.041 | 0.026 | 0.157 | $\phi_{2}^{\max}$ | 4.94 × 10^-3^ | 4.87 × 10^-3^ | 8.07 × 10^-3^ |
| *ξ*_m3_ | 0.021 | 0.020 | 0.164 | $\phi_{3}^{\max}$ | 4.00 × 10^-3^ | 4.00 × 10^-3^ | 4.47 × 10^-3^ |
| *ξ*_m4_ | 0.090 | 0.090 | 0.178 | $\phi_{4}^{\max}$ | 7.24 × 10^-3^ | 7.19 × 10^-3^ | 8.85 × 10^-3^ |
| *ξ*_m5_ | 0.245 | 0.174 | 0.250 | $\phi_{5}^{\max}$ | 7.58 × 10^-3^ | 7.48 × 10^-3^ | 11.0 × 10^-3^ |
| *ξ*_m6_ | 0.116 | 0.083 | 0.237 | $\phi_{6}^{\max}$ | 17.0 × 10^-3^ | 16.1 × 10^-3^ | 17.0 × 10^-3^ |
| *ξ*_m7_ | 0.244 | 0.176 | 0.247 | $\phi_{7}^{\max}$ | 17.0 × 10^-3^ | 16.6 × 10^-3^ | 17.0 × 10^-3^ |
| *ξ*_m8_ | 0.108 | 0.079 | 0.198 | $\phi_{8}^{\max}$ | 6.35 × 10^-3^ | 6.28 × 10^-3^ | 9.17 × 10^-3^ |
| *ξ*_m9_ | 0.020 | 0.020 | 0.248 | $\phi_{9}^{\max}$ | 4.00 × 10^-3^ | 4.00 × 10^-3^ | 4.25 × 10^-3^ |
| *ξ*_m10_ | 0.068 | 0.065 | 0.212 | $\phi_{10}^{\max}$ | 13.2 × 10^-3^ | 13.0 × 10^-3^ | 14.8 × 10^-3^ |
| *ξ*_m11_ | 0.127 | 0.070 | 0.131 | $\phi_{11}^{\max}$ | 5.95 × 10^-3^ | 5.94 × 10^-3^ | 9.00 × 10^-3^ |
| *ξ*_p_ | 7.414 | 6.644 | 9.570 | *τ_G_*_1_ | 270.8 | 258.3 | 309.6 |
| *η*_m1_ | 0.135 | 0.112 | 0.252 | *τ_G_*_2_ | 333.4 | 237.1 | 332.7 |
| *η*_m2_ | 0.360 | 0.223 | 0.405 | *τ_G_*_3_ | 200.1 | 200.0 | 208.4 |
| *η*_m3_ | 0.043 | 0.032 | 0.276 | *τ_G_*_4_ | 337.4 | 292.5 | 347.4 |
| *η*_m4_ | 0.042 | 0.030 | 0.204 | *τ_G_*_5_ | 455.2 | 288.7 | 463.9 |
| *η*_m5_ | 0.030 | 0.030 | 0.152 | *τ_G_*_6_ | 501.2 | 437.4 | 507.5 |
| *η*_m6_ | 0.032 | 0.031 | 0.340 | *τ_G_*_7_ | 599.6 | 505.2 | 600.0 |
| *η*_m7_ | 0.434 | 0.128 | 0.500 | *τ_G_*_8_ | 527.5 | 288.9 | 544.2 |
| *η*_m8_ | 0.031 | 0.030 | 0.172 | *τ_G_*_9_ | 200.1 | 200.0 | 205.9 |
| *η*_m9_ | 0.258 | 0.086 | 0.291 | *τ_G_*_10_ | 598.7 | 509.2 | 600.0 |
| *η*_m10_ | 0.238 | 0.205 | 0.499 | *τ_G_*_11_ | 377.3 | 275.1 | 377.7 |
| *η*_m11_ | 0.205 | 0.193 | 0.291 | *b*_1_ | 5.289 | 4.982 | 5.843 |
| *η*_p_ | 7.064 | 6.815 | 9.927 | *b*_2_ | 9.090 | 7.608 | 9.676 |
| *σ*^*^ | 44.55 | 23.33 | 45.00 | *b*_3_ | 2.521 | 2.500 | 7.648 |
| *z*_0_ | -3.943 | -3.942 | -3.845 | *b*_4_ | 3.971 | 3.932 | 6.959 |
| *z*_1_ | -0.815 | -0.835 | -0.807 | *b*_5_ | 2.544 | 2.500 | 9.548 |
| *c*_1_ | 0.0409 | 0.0400 | 0.0982 | *b*_6_ | 2.504 | 2.500 | 3.147 |
| *c*_2_ | 0.0400 | 0.0400 | 0.1040 | *b*_7_ | 3.133 | 2.928 | 5.582 |
| *c*_3_ | 0.0625 | 0.0574 | 0.1046 | *b*_8_ | 3.071 | 2.872 | 7.805 |
| *c*_4_ | 0.0401 | 0.0400 | 0.1452 | *b*_9_ | 8.097 | 5.608 | 9.979 |
| *c*_5_ | 0.0403 | 0.0400 | 0.1335 | *b*_10_ | 2.600 | 2.556 | 3.275 |
| *c*_6_ | 0.0490 | 0.0465 | 0.1417 | *b*_11_ | 4.195 | 4.190 | 7.015 |
| *c*_7_ | 0.0521 | 0.0521 | 0.0881 | $\mathcal{E}_{1}$ | 992.7 | 674.3 | 1000.0 |
| *c*_8_ | 0.0558 | 0.0543 | 0.1406 | $\mathcal{E}_{2}$ | 576.6 | 340.5 | 609.0 |
| *c*_9_ | 0.0407 | 0.0400 | 0.1493 | $\mathcal{E}_{3}$ | 985.0 | 863.4 | 998.8 |
| *c*_10_ | 0.0726 | 0.0675 | 0.0990 | $\mathcal{E}_{4}$ | 547.9 | 404.4 | 703.7 |
| *c*_11_ | 0.0413 | 0.0400 | 0.1436 | $\mathcal{E}_{5}$ | 823.5 | 478.0 | 941.7 |
| *γ* | 1.200 | 1.200 | 1.766 | $\mathcal{E}_{6}$ | 918.7 | 557.6 | 981.9 |
| *β* | 7.772 | 6.901 | 7.905 | $\mathcal{E}_{7}$ | 1422.9 | 616.5 | 1391.0 |
| ΔH_A_ | -7.45 × 10^4^ | -7.90× 10^4^ | -7.44 × 10^4^ | $\mathcal{E}_{8}$ | 741.5 | 400.7 | 820.6 |
| ΔH_D_ | 4.79 × 10^5^ | 4.16 × 10^5^ | 4.83 × 10^5^ | $\mathcal{E}_{9}$ | 686.7 | 615.6 | 764.9 |
| ΔS_D_ | 601.9 | 600.4 | 1350.2 | $\mathcal{E}_{10}$ | 737.9 | 367.9 | 766.2 |
| *T*_0_ | 6.42 | 6.16 | 6.54 | $\mathcal{E}_{11}$ | 980.9 | 456.1 | 999.9 |
| *ψ*_0_ | -0.80 | -0.82 | -0.79 |  |  |  |  |

Subscript numbers in the symbols refer to tree numbers. See Table 2 for the meanings and units of the symbols.

**Table S3** Maxima a posteriori (MAP) estimates and 99% intervals of the posterior distributions of parameters for site Hyytiälä

|  | MAP | 0.5% | 99.5% |  | MAP | 0.5% | 99.5% |
| --- | --- | --- | --- | --- | --- | --- | --- |
| $\xi_{m}^{Pe15}$ | 0.024 | 0.020 | 0.237 | $\phi_{Pe15}^{\max}$ | 6.20 × 10^-3^ | 5.83 × 10^-3^ | 7.13 × 10^-3^ |
| $\xi_{m}^{Pe16}$ | 0.177 | 0.131 | 0.230 | $\phi_{Pe16}^{\max}$ | 11.8 × 10^-3^ | 11.5 × 10^-3^ | 16.0 × 10^-3^ |
| $\xi_{m}^{Pe17}$ | 0.020 | 0.020 | 0.180 | $\phi_{Pe17}^{\max}$ | 5.85 × 10^-3^ | 5.76 × 10^-3^ | 8.23 × 10^-3^ |
| $\xi_{m}^{Pe18}$ | 0.243 | 0.120 | 0.250 | $\phi_{Pe18}^{\max}$ | 5.68 × 10^-3^ | 4.61 × 10^-3^ | 6.58 × 10^-3^ |
| $\xi_{m}^{Pe19}$ | 0.249 | 0.150 | 0.250 | $\phi_{Pe19}^{\max}$ | 17.0 × 10^-3^ | 16.7 × 10^-3^ | 17.0 × 10^-3^ |
| $\xi_{m}^{Sy15}$ | 0.020 | 0.020 | 0.097 | $\phi_{Sy15}^{\max}$ | 11.7 × 10^-3^ | 11.4 × 10^-3^ | 14.7 × 10^-3^ |
| $\xi_{m}^{Sy17}$ | 0.199 | 0.026 | 0.229 | $\phi_{Sy17}^{\max}$ | 4.00 × 10^-3^ | 4.00 × 10^-3^ | 4.10 × 10^-3^ |
| $\xi_{m}^{Sy18}$ | 0.224 | 0.060 | 0.250 | $\phi_{Sy18}^{\max}$ | 5.37 × 10^-3^ | 4.00 × 10^-3^ | 5.47 × 10^-3^ |
| $\xi_{m}^{Sy19}$ | 0.110 | 0.099 | 0.155 | $\phi_{Sy19}^{\max}$ | 4.01 × 10^-3^ | 4.00 × 10^-3^ | 4.32 × 10^-3^ |
| *ξ*_p_ | 11.986 | 11.944 | 12.298 | $\tau_{G}^{Pe15}$ | 367.4 | 357.2 | 431.7 |
| *z*_0,15_ | -2.908 | -3.077 | -2.872 | $\tau_{G}^{Pe16}$ | 600.0 | 546.8 | 600.0 |
| *z*_1,15_ | -0.404 | -0.506 | -0.386 | $\tau_{G}^{Pe17}$ | 472.9 | 442.0 | 490.0 |
| *z*_0,16_ | -2.456 | -2.582 | -2.451 | $\tau_{G}^{Pe18}$ | 389.6 | 379.2 | 490.7 |
| *z*_1,16_ | -0.201 | -0.338 | -0.200 | $\tau_{G}^{Pe19}$ | 599.7 | 517.1 | 600.0 |
| *z*_0,17_ | -3.048 | -3.202 | -3.007 | $\tau_{G}^{Sy15}$ | 408.7 | 362.6 | 448.4 |
| *z*_1,17_ | -0.497 | -0.654 | -0.479 | $\tau_{G}^{Sy17}$ | 265.7 | 262.0 | 371.2 |
| *z*_0,18_ | -4.235 | -4.254 | -3.808 | $\tau_{G}^{Sy18}$ | 201.5 | 200.0 | 205.6 |
| *z*_1,18_ | -0.995 | -1.000 | -0.827 | $\tau_{G}^{Sy19}$ | 359.8 | 355.9 | 511.4 |
| *z*_0,19_ | -2.832 | -2.955 | -2.785 | *b*_Pe15_ | 3.864 | 2.525 | 6.104 |
| *z*_1,19_ | -0.266 | -0.336 | -0.238 | *b*_Pe16_ | 7.483 | 7.371 | 9.186 |
| *c*_Pe15_ | 0.033 | 0.030 | 0.036 | *b*_Pe17_ | 10.994 | 6.954 | 11.000 |
| *c*_Pe16_ | 0.041 | 0.036 | 0.068 | *b*_Pe18_ | 2.513 | 2.500 | 6.176 |
| *c*_Pe17_ | 0.058 | 0.048 | 0.077 | *b*_Pe19_ | 2.875 | 2.523 | 3.345 |
| *c*_Pe18_ | 0.049 | 0.044 | 0.057 | *b*_Sy15_ | 2.822 | 2.764 | 3.781 |
| *c*_Pe19_ | 0.081 | 0.070 | 0.085 | *b*_Sy17_ | 10.994 | 7.511 | 11.000 |
| *c*_Sy15_ | 0.048 | 0.041 | 0.053 | *b*_Sy18_ | 2.502 | 2.500 | 9.182 |
| *c*_Sy17_ | 0.046 | 0.040 | 0.072 | *b*_Sy19_ | 5.767 | 4.174 | 5.930 |
| *c*_Sy18_ | 0.039 | 0.033 | 0.046 | $\mathcal{E}_{Pe15}$ | 576.5 | 600.0 | 667.1 |
| *c*_Sy19_ | 0.038 | 0.037 | 0.041 | $\mathcal{E}_{Pe16}$ | 1499.0 | 1111.0 | 1500.0 |
| *γ* | 1.600 | 1.600 | 2.510 | $\mathcal{E}_{Pe17}$ | 1496.5 | 894.0 | 1499.7 |
| *β* | 2.560 | 2.470 | 2.743 | $\mathcal{E}_{Pe18}$ | 999.4 | 981.4 | 1434.9 |
| ΔH_A_ | -5.50 × 10^4^ | -6.77 × 10^4^ | -5.41 × 10^4^ | $\mathcal{E}_{Pe19}$ | 1497.4 | 1471.9 | 1500.0 |
| ΔH_D_ | 3.22 × 10^5^ | 2.90 × 10^5^ | 3.77 × 10^5^ | $\mathcal{E}_{Sy15}$ | 602.9 | 600.0 | 1256.0 |
| ΔS_D_ | 772.4 | 735.932 | 1083.715 | $\mathcal{E}_{Sy17}$ | 1495.9 | 1197.5 | 1500.0 |
| *T*_0_ | 5.64 | 5.13 | 5.66 | $\mathcal{E}_{Sy18}$ | 605.1 | 600.1 | 896.2 |
| *ψ*_0_ | -1.10 | -1.10 | -1.09 | $\mathcal{E}_{Sy19}$ | 788.8 | 736.6 | 988.8 |

Superscript or subscript ‘Pe’ and ‘Sy’ refer to trees Pentti and Sylvi, and 15—19 years 2015—2019, respectively. See Table 2 for the meanings and units of the symbols.

**Fig. S1** Modelled and observed transpiration rate (*E*) of the four Norway spruce trees in Fig. 1 & 2 at the peatland site (PS). Each panel title displays the tree number, treatment of the block (C, control; SH, select harvest), and tree height. The grey stripes, the same as in Fig. 1, correspond to the period in a zoom-in view displayed in Fig. 2.

**Fig. S2** Modelled and observed transpiration rate (*E*) of Scots pine tree ’Pentti’ at the mineral-soil site (MS) over 2015—2019. The grey stripes, the same as in Fig. 3, correspond to the period in a zoom-in view displayed in Fig. 4.

**Fig. S3** Modelled and observed transpiration rate (*E*) and stem radial dimension (SRD) change in the growing season (*d* – *d*_0_) of the other Norway spruce trees at the peatland site than those in Fig. 1 & 2. For each tree, the first two rows of panels (*E* and *d* – *d*_0_ at 30-minute resolution) are the zoom-in view that correspond to the grey stripes in the third (daily-mean *E* of the days with ≥ 70% data available) and fourth (*d* – *d*_0_ and precipitation at 30-minute resolution) rows, respectively. FM, AM1 and AM2 are full model, alternative models without phenology (Gompertz function) and using only the Gompertz function for modelling growth, respectively. SRD dynamics due to hydraulics modelled by AMs are not shown for clarity.

**Fig. S4** Modelled and observed transpiration rate (*E*) and stem radial dimension change (SRD) in growing season (*d* – *d*_0_) of Scots pine tree “Sylvi” at the mineral-soil site. Data of 2016 were unavailable for technical failure of measurements. The arrangement of panels, legend and the meanings of FM, AM1 and AM2 are the same as in Fig. S1, except results are separated by the year (cf. tree). SRD dynamics due to hydraulics modelled by AMs are not shown for clarity.
